# DDX17 induces epithelial-mesenchymal transition and metastasis through the miR-149-3p/CYBRD1 pathway in colorectal cancer

**DOI:** 10.1038/s41419-022-05508-y

**Published:** 2023-01-02

**Authors:** Gang Zhao, Qijing Wang, Yue Zhang, Rui Gu, Min Liu, Qin Li, Jie Zhang, Hang Yuan, Tianyu Feng, Deqiong Ou, Siqi Li, Shan Li, Kai Li, Chunfen Mo, Ping Lin

**Affiliations:** 1 Lab of Experimental Oncology, State Key Laboratory of Biotherapy and Cancer Center, and Frontiers Science Center for Disease-related Molecular Network, West China Hospital, Sichuan University, Chengdu, Sichuan Province China; 2 Department of General Surgery, Second Affiliated Hospital of Chengdu Medical College (China National Nuclear Corporation 416 Hospital), Chengdu, Sichuan Province China

**Keywords:** Cell migration, Metastasis

## Abstract

DEAD box helicase 17 (DDX17) has been reported to be involved in the initiation and development of several cancers. However, the functional role and mechanisms of DDX17 in colorectal cancer (CRC) malignant progression and metastasis remain unclear. Here, we reported that DDX17 expression was increased in CRC tissues compared with noncancerous mucosa tissues and further upregulated in CRC liver metastasis compared with patient-paired primary tumors. High levels of DDX17 were significantly correlated with aggressive phenotypes and worse clinical outcomes in CRC patients. Ectopic expression of DDX17 promoted cell migration and invasion in vitro and in vivo, while the opposite results were obtained in DDX17-deficient CRC cells. We identified miR-149-3p as a potential downstream miRNA of DDX17 through RNA sequencing analysis, and miR-149-3p displayed a suppressive effect on the metastatic potential of CRC cells. We demonstrated that CYBRD1 (a ferric reductase that contributes to dietary iron absorption) was a direct target of miR-149-3p and that miR-149-3p was required for DDX17-mediated regulation of CYBRD1 expression. Moreover, DDX17 contributed to the metastasis and epithelial to mesenchymal transition (EMT) of CRC cells via downregulation of miR-149-3p, which resulted in increased CYBRD1 expression. In conclusion, our findings not only highlight the significance of DDX17 in the aggressive development and prognosis of CRC patients, but also reveal a novel mechanism underlying DDX17-mediated CRC cell metastasis and EMT progression through manipulation of the miR-149-3p/CYBRD1 pathway.

## Introduction

Colorectal cancer (CRC) is the third most commonly diagnosed cancer and the second leading cause of cancer death worldwide, with an estimated 1.9 million new cancer cases and 0.9 million cancer deaths in 2020 [1]. Although the majority of patients diagnosed with early-stage CRC can be cured by surgery, the 5-year survival declines to 12% for stage IV disease, which is caused by distant tumor metastasis [2]. Advances in surgical techniques and innovative targeted therapeutic combinations over the past two decades have effectively prolonged the median survival of patients with metastatic CRC [3, 4]. Accumulating evidence has indicated that CRC is a highly heterogeneous disease characterized by extensively dysregulated gene expression and mutational burdens [5, 6]. For instance, the adenomatous polyposis coli (APC) gene is frequently mutated in CRC and inactivation of APC has been considered to assist the initial development of CRC from normal epithelial cells [7]. Constitutive activation of the oncogene KRAS or BRAF leads to malignant transformation and progression of CRC through enhanced cell proliferation, anti-apoptosis, angiogenesis, invasion, and metastasis [8]. In addition, genetic loss of PTEN, which counteracts the PI3K/Akt signaling cascade, facilitates the continuous growth of CRC cells and is associated with worse outcomes in CRC patients [9]. Thus, a better understanding of the malignant progression of CRC will be helpful for the development of potential targets and therapeutic opportunities.

DEAD-box helicase 17 (DDX17), which is a multifunctional ATP-dependent RNA/DNA helicase and a cofactor of the Drosha-DGCR8 complex, has been shown to play vital roles in eukaryotes, including pre-mRNA alternative splicing, miRNA processing and transcriptional regulation [10, 11]. Recently, increasing evidence has been reported that DDX17 is involved in the initiation and development of several tumors. For example, DDX17 induces the retention of PXN-AS1 intron 3 to produce an oncogenic isoform PXN-AS1-IR3 transcript, which activates MYC transcription and promotes hepatocellular carcinoma metastasis [12]. DDX17 directly bound and augmented Sox2-mediated stem-like features in ER-positive breast cancer [13]. DDX17 is highly expressed in prostate cancer tissues and is essential for the proliferation and migration of prostate cancer cells [14]. However, the function of DDX17 in CRC metastasis remains unknown.

MicroRNAs (miRNAs) are endogenous small noncoding RNAs of 18–24 nucleotides in length that directly bind to the 3′ untranslated regions (UTRs) of mRNAs, leading to the degradation and translational suppression of target genes [15, 16]. MiR-149-3p is generated from the precursor miR-149 gene, which is located at chromosome 2q37.3. Numerous studies have demonstrated that dysregulation of miR-149-3p contributes to various pathological processes of cancers, including tumor cell metastasis, proliferation and apoptosis. For instance, miR-149-3p diminishes glucose metabolism and strengthens 5-FU-induced cell death by targeting PDK2 in CRC [17]. The increased level of miR-149-3p caused by dioscin significantly inhibits pancreatic cancer cell growth via blockage of Akt1 signaling [18]. Moreover, miR-149-3p decreases Wnt-1 expression and acts as a tumor suppressor in gastric cancer [19]. A recent study suggested a potential role of miR-149-3p in CRC metastasis [20]. However, the detailed molecular mechanism of miR-149-3p in aggressive CRC progression needs to be further explored.

In the present study, we investigated the functional role of DDX17 in CRC metastasis and its prognostic significance in patients with CRC. We found that DDX17 expression was higher in CRC tissues than in noncancerous mucosal tissues and was further upregulated in CRC liver metastases compared with patient-paired primary tumors. Elevated DDX17 levels were positively associated with aggressive phenotypes and poor prognosis of CRC patients. DDX17 overexpression enhanced metastatic and invasive potential by facilitating EMT induction in CRC cells. Mechanistically, we identified that DDX17 regulated gene expression through miRNA-mediated mechanisms and that DDX17 contributed to CRC metastasis through downregulation of miR-149-3p. We further demonstrated that CYBRD1 (a ferric reductase that contributes to dietary iron absorption) was a direct target of miR-149-3p. Taken together, our findings indicated that DDX17 promoted metastasis and EMT progression in CRC through modulation of the miR-149-3p/CYBRD1 pathway, implicating DDX17 as a valuable predictor of prognosis in CRC patients and a potential therapeutic target for CRC.

## Materials and methods

### Cell culture

Human CRC cell lines (DLD1, HCT116, HCT15, SW480, SW620, LoVo, and HT29) were obtained and authenticated by short tandem repeat profiling from the Chinese Academy of Sciences Cell Bank (Shanghai, China). All cells were cultured in Dulbecco’s modified Eagle’s medium (DMEM, Gibco) with 10% fetal bovine serum (Gibco) and 100 μg/ml penicillin/streptomycin at 37 °C in a 5% CO_2_ incubator. Cells were tested for mycoplasma contamination every 6 months and confirmed to be negative.

### Human clinical samples and immunohistochemical analysis

Clinical specimens in this study (cohort I), including CRC tissues (n = 133) and adjacent normal tissues (*n* = 69), from CRC patients who underwent surgical resection from 2008 to 2013 were obtained from West China Hospital. Cohort 2 contained 28 paired CRC liver metastasis and matched primary CRC tissues. All patients were of Han nationality and provided signed informed consent. This study was approved by the Ethics Committee on Biomedical Research, West China Hospital of Sichuan University (2021(594)) and performed in accordance with the Helsinki Declaration of 1975. All animal experiments were performed in accordance with relevant guidelines and regulations, and approved by the Institutional Animal Care and Use Committee of West China Hospital (2021087 A). The clinical pathological information of HCC patients is summarized in Table 1. The expression of DDX17 in CRC tissues was determined by immunohistochemical analysis as described previously [21]. Each staining was independently evaluated by two pathologists, and the investigators were blinded to group allocation during the experiment.

**Table 1:** The correlation of DDX17 expression and clinicopathological characteristics of CRC patients.

Parameters	Cases (*n* = 133)	DDX17 expression		χ^2^	*P* values
		Low (*n* = 66)	High (*n* = 67)		
Age (years)				0.366	0.545
<65	68	32	36		
≥65	65	34	31		
Gender				0.361	0.584
Female	55	29	26		
Male	78	37	41		
Tumor location				4.032	0.133
Right colon	32	11	21		
Left colon	28	16	12		
Rectum	73	39	34		
Tumor size				3.980	0.046
<5 cm	67	39	28		
≥5 cm	66	27	39		
Histological grade				3.668	0.160
Well	39	24	15		
Moderate	79	34	45		
Poor	15	8	7		
Tumor invasion				4.268	0.118
T1 + T2	10	7	3		
T3	117	58	59		
T4	6	1	5		
Lymph node invasion				55.172	<0.001
Absent	74	58	16		
Present	59	8	51		
Distant metastasis				5.185	0.023
Absent	123	65	58		
Present	10	1	9		
AJCC stage				56.454	<0.001
Stage I	6	6	0		
Stage II	68	52	16		
Stage III	49	7	42		
Stage IV	10	1	9		

### Lentivirus infection and transfection

The DDX17 cDNA (NM_006386.4) fragment was inserted into the lentiviral GV248 vector (Genechem Company). Two short hairpin RNAs (shRNAs) targeting DDX17 (DDX17 shRNA#1: 5′-CAAGGGUACCGCCUAUACC-3′; and DDX17 shRNA#2: 5′-GAGAGACUCUGCAAGCUAU-3′) and negative control (shNC: 5′-UUCUCCGAACGUGUCAGGU-3) were cloned into the lentiviral GV358 vector (Genechem Company), respectively. The packaging and purification of lentivirus were performed by Genechem Company (Shanghai, China). CRC cells with stable DDX17 deficiency or overexpression were selected with puromycin for 1-2 weeks. Human miR-149-3p mimic (5′-AGGGAGGGACGGGGGCUGUGC-3′), miR-149-3p antisense (5′-GCACAGCCCCCGUCCCUCCCU-3′) and CYBRD1 shRNA (5′-UCCAUCCAUGCAGGGUUAAAU-3′) were inserted into the lentiviral LV-3 vector and the lentivirus was packaged by Genepharma Company (Shanghai, China). Small interfering RNAs (siRNAs) targeting CYBRD1 and DDX5 were purchased from Thermo Fisher Scientific. SiRNA was transfected into CRC cells using Lipofectamine RNAiMAX (Thermo Fisher Scientific) according to the manufacturer’s instructions.

### RNA extraction and RT‒qPCR

Total RNA was extracted from the cells using TRIzol reagent (Invitrogen) according to the manufacturer’s instructions. Reverse transcription was performed with PrimeScript RT Reagent Kit (Takara Biomedical Technology) for mRNA or All-in-One™ miRNA First-Strand cDNA Synthesis Kit (Genecopoeia) for miRNA according to the manufacturer’s instructions. Quantitative PCR was performed with TB Green^®^ Premix Ex Taq™ II (Takara Biomedical Technology) for mRNA or All-in-One™ miRNA qRT‒PCR Detection Kits (Genecopoeia) for miRNA using a CFX Connect real-time PCR system (Bio-Rad). The relative expression levels of miRNA and mRNA were calculated by the 2^−∆∆Cq^ method and normalized to the internal controls U6 and 18S, respectively. The primers for pri-miR-149, pre-miR-149, miR-149-5p and miR-149-3p were purchased from Genecopoeia, and the primers for mRNA were as follows: DDX17 forward: 5′-GATGAAGAAGGAGGCGGAGAAGAG-3′ and reverse: 5′-GGGGTCAGTATCAGCCGCTTTCAG-3′; DDX5 forward: 5′-CGGGACCGAGGGTTTGGTG-3′ and reverse: 5′-GCAGCTCATCAAGATTCCACTTC-3′; CYBRD1 forward: 5′-GCAACAGCACTTATGGGATTG-3′ and reverse: 5′-CATTGCGGTCTGGTGACTATC-3′; 18S forward: 5′-TTGACGGAAGGGCACCACCAG-3′ and reverse: 5′-GCACCACCACCCACGGAATCG-3′.

### RNA sequencing analysis

Total RNA was extracted from the indicated CRC cells using TRIzol reagent (Invitrogen). For RNA-sequencing, total RNA was enriched by using oligo(dT)-attached magnetic beads, fragmented and reverse transcribed to cDNA. cDNAs were subjected to adaptor ligation and PCR amplification. The paired-end sequencing (PE150) of the cDNA library was carried out on an Illumina HiSeq^TM^ 3000 platform by RiboBio Co. Ltd. (Guangzhou, China). For miRNA-sequencing, total RNA was ligated with adaptors, reverse transcribed and amplified by PCR. Single-end sequencing (SE50) of the cDNA library with an insert fragment between 18 nt and 40 nt was carried out on an Illumina HiSeq^TM^ 2500 platform by RiboBio Co. Ltd. (Guangzhou, China). Clean reads were aligned to the human genome (release hg19) using Tophat2 (for RNA) or Bowite 2.2.5 (for miRNA) software with default parameters. The expression levels of mRNA and miRNA were calculated as RPKM (Reads Per Kilobase per Million mapped reads) and RPM (Reads Per Million clean tags), respectively. The differentially expressed genes and miRNAs with three biological replicates were analyzed by RiboBio Co. Ltd. (Guangzhou, China) using DEGseq and edgeR software, respectively.

### Cell migration and invasion assays

Cell migration and invasion assays were carried out in Transwell chambers (8 μm pore size, Corning). For the invasion assay, the chambers were precoated with growth-factor-reduced Matrigel (Sigma). CRC cells resuspended in 200 μL serum-free DMEM medium were added to the upper chambers, while the bottom chambers were filled with DMEM medium supplemented with 10% fetal bovine serum. After 48 h of incubation, the cells in the chamber were fixed with 4% paraformaldehyde for 20 min and stained with 0.1% crystal violet for 15 min. The cells were observed and counted in five randomly selected fields under a microscope. The numbers of migratory and invasive cells were photographed by a microscope and counted using ImageJ software.

### Cell adhesion assays

Cell adhesion assays were performed as described previously [22]. Briefly, the 96-well plate was precoated with 50 μL Matrigel (Sigma) for 2 h. Then, 100 μL CRC cell suspensions (1 × 10^4^ cells per well) were plated into the covered wells and incubated for 30, 60, 90, and 120 min at 37 °C. The unadhered cells were washed away with PBS buffer. The number of adhered cells was measured as the average of five random fields in each well under a microscope (×100 magnification), and each group included three replications.

### Luciferase reporter assay

The luciferase reporter assay was carried out as described previously [23]. Briefly, dual-luciferase reporter plasmids PEZx-FR02 (Genecopoeia) inserted with a fragment of the CYBRD1 3′-UTR containing putative or mutated binding sites of miR-149-3p were cotransfected into HEK293T cells along with miR-149-3p mimic or mimic control by Lipofectamine 3000 (Thermo Fisher Scientific). After 36 h posttransfection, the relative luciferase activity was analyzed by the Dual-Luciferase Reporter Assay System (Promega Corporation) using a MultiMode Microplate Reader (Synergy 2, BioTek) following the manufacturer’s protocols.

### Western blot and immunoprecipitation

CRC cells were lysed in RIPA buffer (50 mM Tris-HCl pH 7.5, 1% NP-40, 0.25% sodium deoxycholate and 150 mM NaCl) supplemented with protease inhibitors (TargetMol, C0001). Lysates were loaded and separated by SDS–PAGE, transferred to a PVDF membrane (Millipore), and then incubated with the following antibodies: anti-DDX17 (Santa Cruz Biotechnology, sc-398168), anti-DDX5 (Cell Signaling Technology, CST, #9877), anti-CYBRD1 (Abcam, ab66048), anti-E-cadherin (CST, #3195), anti-Claudin-1 (CST, #13255), anti-Vimentin (CST, #5741), anti-N-cadherin (CST, #13116), and anti-β-actin (Sangon, D110001). On the next day, the membrane was probed with secondary antibodies at room temperature for 1 h, and protein bands were visualized using Immobilon™ Western Chemiluminescent HRP Substrate (Millipore). For RNA immunoprecipitation, pri-miR-149 and pri-miR-21 were in vitro transcribed and biotinylated by using T7 RNA polymerase and RNA 3’ End Desthiobiotinylation Kit (Thermo Fisher Scientific) according to the manufacturer’s instructions, respectively. The biotinylated pri-miRNA was incubated with protein lysates from SW620 cells, immunoprecipitated with streptavidin beads and then subjected to western blot analysis by using anti-DDX17 antibody. Pri-miR-21 has been demonstrated to bind with DDX17 [24] and thus was used as a positive control.

### Intracellular iron assay

CRC cells were collected and washed twice with ice-cold PBS, and then rapidly homogenized in 4–10 volumes of Iron Assay buffer. The intracellular iron content was quantified by using an Iron Assay Kit (Sigma–Aldrich, MAK025) following the manufacturer’s protocols.

### In vivo assays for tumor metastasis

Female BALB/c nude mice (6-week old) were obtained from Gempharmatech Company (Jiangsu, China) and raised in specific pathogen-free conditions. SW620 cells stably expressing shDDX17 or shNC and SW480 cells stably expressing DDX17 or mock were used to explore the function of DDX17 in tumor metastasis. To assess the effect of miR-149-3p on DDX17-mediated CRC malignant progression, SW480 cells were infected with DDX17 cDNA lentivirus and miR-149-3p mimic lentivirus. To evaluate the impact of CYBRD1 on miR-149-3p-mediated CRC development, SW480 cells were infected with miR-149-3p inhibitor lentivirus and CYBRD1 shRNA lentivirus. The above CRC cells were infected with lentivirus stably expressing luciferase (pLV-Neo-CMV-luciferase, Yunzhou Biosciences, China). The mouse liver metastatic model was established as described previously [25]. Briefly, mice were randomly divided into the corresponding groups. The sample size (*n* = 6) was determined based on previous experience, which was used to compensate for higher natural variance in vivo. Mice were anesthetized by pentobarbital sodium (50 mg/kg, intraperitoneally), and the spleens of mice were exposed with a small abdominal incision. A total of 1 × 10^6^ CRC cells were suspended in 50 μL PBS and injected into the spleens of mice. Then, the incisions were stitched, and the mice were raised for 2 months. For luciferase intensity detection, mice were intraperitoneally injected with D-luciferin (150 mg/kg). Ten minutes later, the mice were euthanized, and the liver tissues were removed to detect light emission by an IVIS Spectrum in vivo imaging system. No data were excluded from our analyses. All animal experiments were approved by the Institutional Animal Care and Use Committee (IACUC) of West China Hospital Sichuan University.

### Statistical analysis

All experiments were performed independently with at least three biological replicates, and all data are displayed as the mean ± SD. The sample size was determined based on previous studies or papers, which would allow for adequate analysis to achieve valuable conclusions from the data. Student’s *t* test was applied to compare two groups, and one-way ANOVA was used for the comparison of more than two groups. Pearson’s χ^2^ test was used for the correlation analysis. Survival analysis was evaluated by the Kaplan–Meier method. A multivariate Cox proportional hazards model was used to assess the independent prognostic factors for overall survival. Statistical analysis was performed using SPSS 22.0 (IBM Corp) and Prism 6 (GraphPad Software). *P* < 0.05 was considered statistically significant.

## Results

### DDX17 is upregulated in CRC and correlates with CRC liver metastasis

**Fig. 1:**
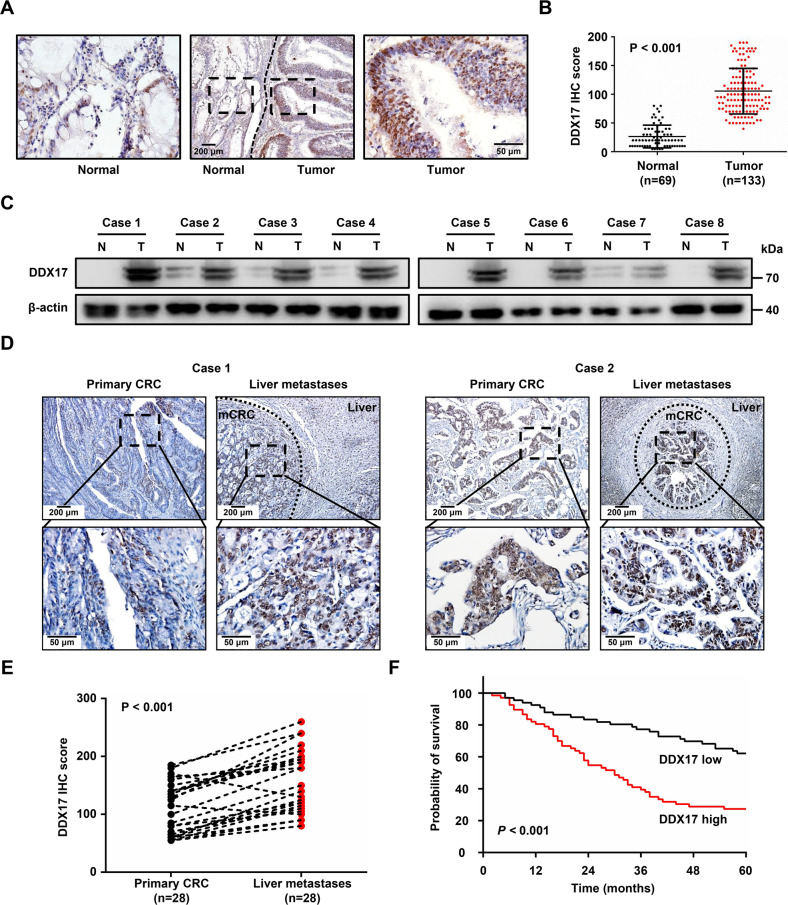
DDX17 is upregulated in CRC and correlates with CRC liver metastasis. **A** A representative immunohistochemical staining image of DDX17 in human CRC tissue. **B** Relative DDX17 protein expression in 69 normal mucosa tissue samples and 133 CRC tissue samples. **C** Western blot analysis of DDX17 levels in CRC tissues and patient-matched adjacent normal tissues. **D** Two representative immunohistochemical staining images of DDX17 in CRC liver metastasis and patient-matched primary CRC samples. **E** Relative DDX17 protein expression in 28 pairs of primary CRC and CRC liver metastasis tissues. **F** Kaplan–Meier analysis of the overall survival of CRC patients based on DDX17 protein levels.

To investigate the role of DDX17 in the development of CRC, we first evaluated the mRNA level of DDX17 in CRC tissues using TCGA database. We found that DDX17 mRNA expression was increased in colon adenocarcinoma (COAD) tissues compared with normal tissues (Supplementary Fig. S1A). Next, we determined the protein expression of DDX17 in 69 normal mucosa tissue samples and 133 CRC tissue samples. Immunohistochemical staining showed that DDX17 protein was significantly upregulated in CRC tissues compared with noncancerous mucosa tissues (Fig. 1A, B). Western blot analysis demonstrated that DDX17 levels were higher in CRC tissues than in paired normal mucosa tissues (Fig. 1C). A consistent result was observed in the Clinical Proteomic Tumor Analysis Consortium (CPTAC) colon cancer database (Supplementary Fig. S1B). We used the median of DDX17 expression as a cutoff value and found that elevated DDX17 protein expression was positively associated with larger tumor size, lymph node invasion, distant organ metastasis and advanced AJCC stage, suggesting that DDX17 may be involved in the metastatic progression of CRC (Table 1). To further assess the correlation of DDX17 and CRC metastasis, we examined the expression pattern of DDX17 protein in 28 samples of CRC liver metastatic tissues with patient-matched primary tumor tissues. Stronger DDX17 staining was identified in CRC liver metastasis tissues than in primary CRC tissues (Fig. 1D, E). Subsequently, we analyzed the prognostic significance of DDX17 in CRC. Kaplan–Meier survival analysis revealed that CRC patients with a high level of DDX17 had a shorter overall survival (Fig. 1F). Multivariate analysis showed that DDX17 expression, lymph node invasion and AJCC stage were independent prognostic factors for overall survival in CRC patients (Table 2). Collectively, our results indicate that DDX17 is upregulated in CRC, particularly in CRC liver metastatic lesions, suggesting that DDX17 may contribute to aggressive progression and metastasis in CRC.

**Table 2:** Univariate and multivariate analysis of factors associated with survival in CRC.

Characteristics	Univariate analysis			Multivariate analysis		
	HR	95%CI	*P*	HR	95%CI	*P*
DDX17 (Low vs. High)	0.276	0.167–0.456	<0.001	0.401	0.222–0.722	0.002
Age (<65 vs. ≥65)	1.410	0.888–2.241	0.146			
Gender (Female vs. Male)	0.945	0.591–1.510	0.814			
Tumor size (<5 cm vs. ≥5 cm)	0.624	0.393–0.991	0.046			
Histological grade (Well/moderate vs. Poor)	0.617	0.339–1.124	0.115			
Tumor invasion (T1-T3 vs. T4)	0.433	0.174–1.078	0.072			
Lymph node invasion (Absent vs. Present)	0.594	0.375–0.942	0.027	0.597	0.375–0.951	0.030
Distant metastasis (Absent vs. Present)	0.622	0.285–1.356	0.232			
AJCC stage (I–II vs. III–IV)	0.282	0.174–0.457	<0.001	0.466	0.265–0.818	0.008

### DDX17 enhances CRC cell migration and invasion in vitro and in vivo

**Fig. 2:**
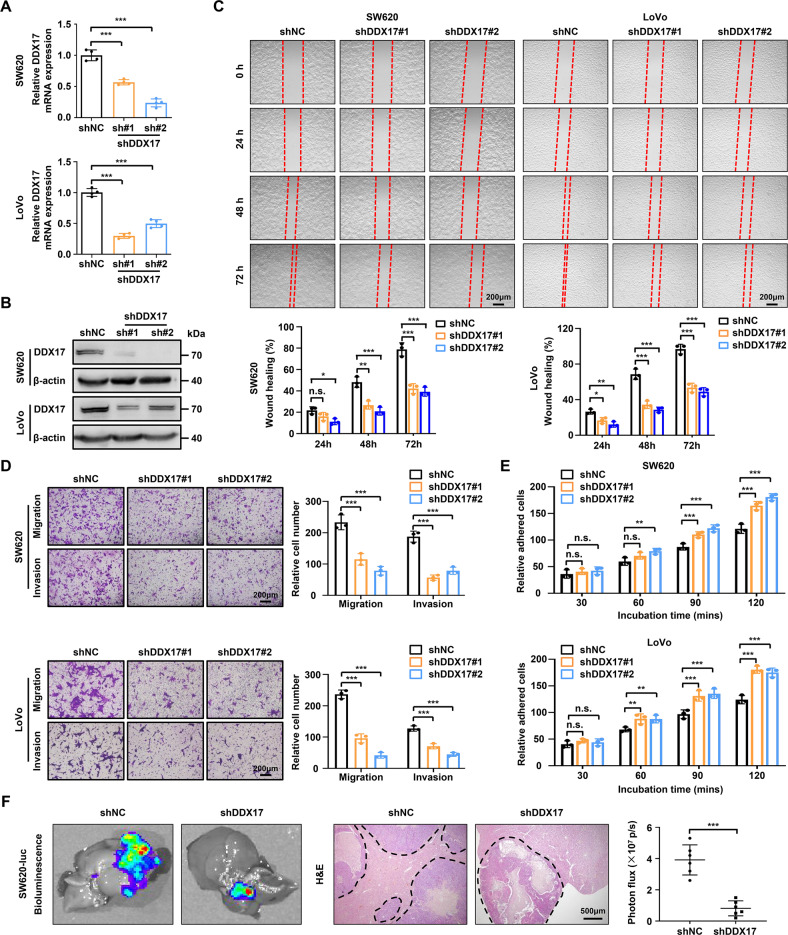
DDX17 silencing suppresses the migration and invasion of CRC cells in vitro and in vivo. **A**, **B** RT‒qPCR (**A**) and western blot (**B**) analysis verified the downregulation of DDX17 mediated by two specific shRNAs targeting DDX17 in SW620 and LoVo cells. **C** Microscopic observations were recorded at 0, 24, 48, and 72 h after scratching the surface of a confluent layer of the indicated SW620 and LoVo cells. **D** The effects of DDX17 on cell migration and invasion were examined by transwell assays in SW620 and LoVo cells. **E** The numbers of adhesive SW620 and LoVo cells on the Matrigel were recorded after 30, 60, 90, and 120 min. **F** DDX17-deficient and control SW620 cells were injected into the spleens of nude mice. Representative bioluminescent and H&E staining images of the isolated liver tissues were obtained, and the light emissions were quantified. n.s. no significance, **P < 0.01, ***P < 0.001.

To explore the function of DDX17 in CRC metastasis, we first determined the expression of DDX17 in seven CRC cell lines. DDX17 was obviously increased in highly metastatic LoVo and SW620 cells compared with weakly metastatic CRC cells (HCT116, HCT15, HT29, DLD1, and SW480) (Supplementary Fig. S2A). We also interrogated the expression of DDX17 mRNA in a series of colorectal cancer cell lines from the CCLE dataset. Although there was no statistical difference in the overall expression status of the DDX17 transcript between highly and weakly metastatic CRC cells, LoVo and SW620 cells had higher DDX17 mRNA levels than HCT116, HT29, DLD1, and SW480 cells (Supplementary Fig. S2B). Next, we established stable shRNA-mediated knockdown of DDX17 in SW620 and LoVo cells with higher DDX17 levels and stable upregulation of DDX17 expression in SW480 and HCT116 cells with lower DDX17 levels. The knockdown and overexpression efficiency of DDX17 were confirmed by RT‒qPCR and western blotting (Fig. 2A, B and Supplementary Fig. S3A, B). DDX17 knockdown resulted in markedly delayed wound closure, whereas ectopic expression of DDX17 notably increased the wound healing percentage compared with that of the control group (Fig. 2C and Supplementary Fig. S3C). A similar result was validated by transwell assays (Fig. 2D and Supplementary Fig. S3D). Importantly, downregulation of DDX17 enhanced the adhesion to extracellular matrix in SW620 and LoVo cells, while DDX17 overexpression decreased the adhesion abilities of SW480 and HCT116 cells (Fig. 2E and Supplementary Fig. S3E). To further verify whether DDX17 was involved in the metastasis of CRC cells in vivo, we established a mouse liver metastasis model by injecting CRC cells into the spleens of nude mice. As shown in Fig. 2F, we observed a significant decrease in liver metastatic lesions in DDX17-deficient SW620 cells. In contrast, DDX17 overexpression augmented the metastatic ability of SW480 cells compared with mock SW480 cells (Fig. S3F). In addition, we evaluated the effect of DDX17 downregulation on the proliferation of CRC cells. The results from the CCK8 assay and colony formation experiment showed that suppression of DDX17 diminished cell viability or anchorage-dependent cell growth in SW620 and LoVo cells (Supplementary Fig. S4). Taken together, these data imply that DDX17 contributes to tumor metastasis in CRC.

### DDX17 promotes epithelial-mesenchymal transition (EMT) in CRC

**Fig. 3:**
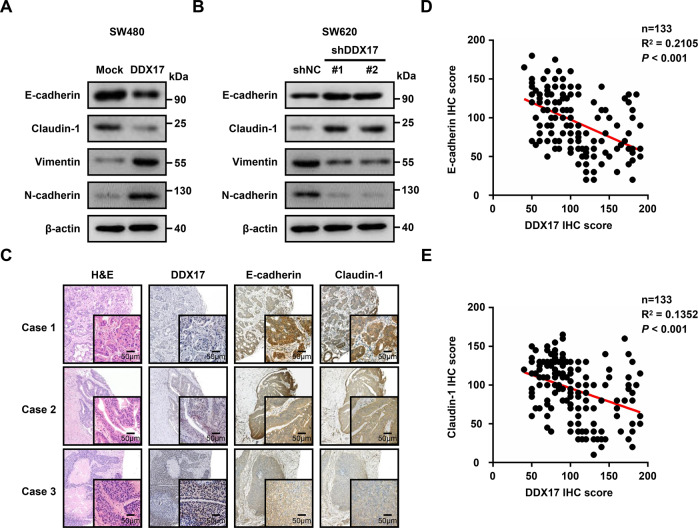
DDX17 induces EMT progression in CRC. **A** Western blot analysis of EMT marker expression in DDX17-overexpressing and mock SW480 cells. **B** Western blot analysis of EMT marker expression in DDX17-deficient and control SW620 cells. **C** Representative immunohistochemical staining images of DDX17, E-cadherin, and Claudin-1 levels in CRC tissues. **D** Correlation analysis between the protein expressions of DDX17 and E-cadherin in CRC tissues. **E** Correlation analysis between the protein expressions of DDX17 and claudin-1 in CRC tissues.

Considering the critical role of the EMT process in tumor metastasis, we examined the expression of epithelial markers (E-cadherin and claudin-1) and mesenchymal markers (N-cadherin and vimentin) to address whether DDX17 is required for the EMT progression of CRC. Exogenous expression of DDX17 in SW480 cells resulted in EMT-like changes, as evidenced by decreased levels of Claudin-1 and E-cadherin as well as increased levels of Vimentin and N-cadherin (Fig. 3A). Conversely, silencing DDX17 in SW620 cells resulted in the opposite effects (Fig. 3B). Next, we evaluated the potential correlation between DDX17 and EMT in clinical CRC tissues. Immunohistochemical analysis revealed that CRC patients with lower DDX17 levels exhibited higher expression levels of E-cadherin and claudin-1 (Fig. 3C). Moreover, DDX17 protein expression was negatively associated with E-cadherin (*R*^2^ = 0.2105, *P* < 0.001) and Claudin-1 (*R*^2^ = 0.1352, *P* < 0.001) expression (Fig. 3D, E). These results indicate that DDX17 promotes EMT progression in CRC.

### MiR-149-3p inhibited the migration and invasion of CRC cells

**Fig. 4:**
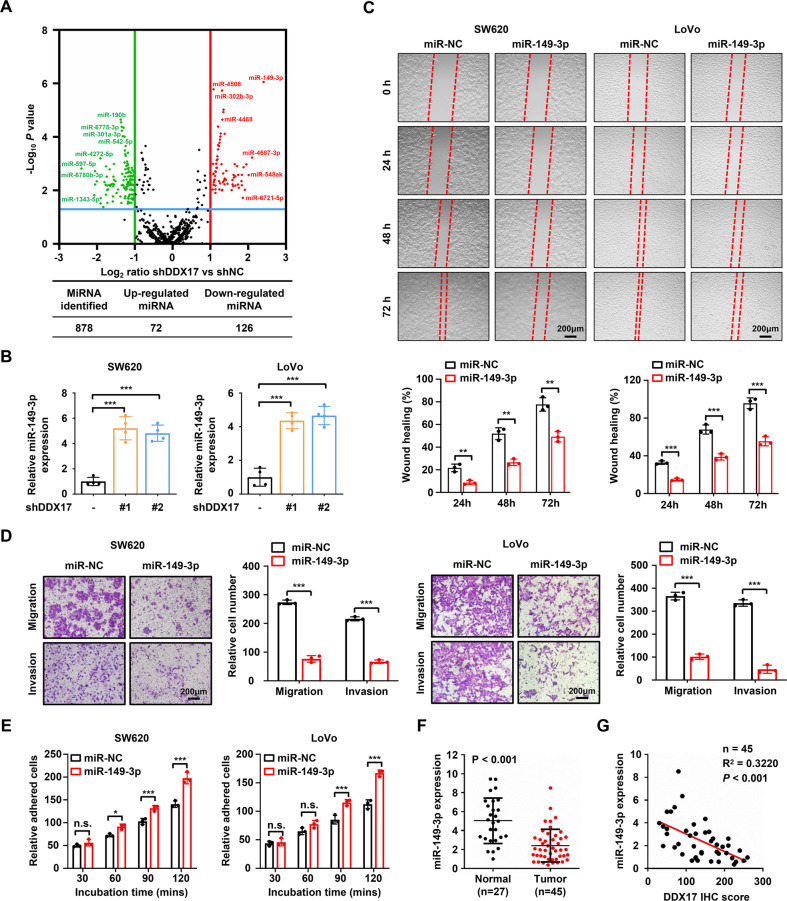
MiR-149-3p inhibited the migratory and invasive abilities of CRC cells. **A** Volcano plot of dysregulated miRNAs in response to DDX17 knockdown. The twofold upregulated and downregulated miRNAs with adjusted *p* value < 0.05 are marked by red and green, respectively. **B** RT‒qPCR determined the impacts of DDX17 on miR-149-3p expression in SW620 and LoVo cells. **C** Microscopic observations were recorded at 0, 24, 48, and 72 h after scratching the surface of a confluent layer of the indicated SW620 and LoVo cells. **D** The effects of miR-149-3p on cell migration and invasion were examined by transwell assay in SW620 and LoVo cells. **E** The numbers of adhesive cells on the Matrigel were recorded after 30, 60, 90, and 120 min in the indicated SW620 and LoVo cells. **F** Relative miR-149-3p levels determined by RT‒qPCR analysis in 27 samples of normal mucosa tissues and 45 samples of CRC tissues. **G** Correlation analysis between DDX17 protein expressions and miR-149-3p levels in CRC tissues. n.s. no significance, *P < 0.05, **P < 0.01, ***P < 0.001.

DDX17 is a cofactor associated with the Microprocessor complex that recognizes and cleaves the primary miRNA (pri-miRNA) transcript to generate pre-miRNA (~70 nucleotide stem–loop intermediate), and pre-miRNA is further processed by the Dicer complex to form mature miRNA duplexes [24, 26]. Thus, we performed miRNA sequencing analysis to assess the abnormal expression of microRNAs caused by DDX17 in CRC cells. We identified 72 upregulated miRNAs and 126 downregulated miRNAs between DDX17-deficient and control SW620 cells by using a criterion of twofold change and adjusted *p* value < 0.05 (Fig. 4A and Supplementary Table S1). As miR-149-3p was the most significantly changed among the dysregulated miRNAs, we selected miR-149-3p for further investigation. Consistent with the RNA sequencing results, the RT‒qPCR assay demonstrated that DDX17 suppression caused notably increased expression of miR-149-3p in SW620 and LoVo cells (Fig. 4B). Because DDX5 is a physical partner of DDX17, we further evaluated the effect of DDX5 on the expression of miR-149-3p in CRC cells. Our results showed that no significant difference in miR-149-3p levels was observed in DDX5-deficient CRC cells (Supplementary Fig. S5). To explore whether pri-miR-149 could directly bind to DDX17, pri-miR-149 was in vitro transcribed and labeled with biotin, and then incubated with protein lysates from SW620 cells. Immunoprecipitation assay illustrated that pri-miR-149 did not precipitate DDX17 (Supplementary Fig. S6A). Moreover, DDX17 was unable to affect the expressions of pri-miR-149, pre-miR-149 and miR-149-5p (Supplementary Fig. S6B), implying that the regulation of miR-149-3p by DDX17 is independent of its Microprocessor function. Next, SW620 and LoVo cells were infected with miR-149-3p lentivirus to assess whether miR-149-3p was involved in the aggressive progression of CRC cells (Supplementary Fig. S7). MiR-149-3p overexpression diminished the migration and invasion of SW620 and LoVo cells, as determined by wound healing and transwell assays (Fig. 4C, D). In addition, upregulated miR-149-3p accelerated cell adhesion of SW620 and LoVo cells (Fig. 4E). We examined miR-149-3p expression in CRC samples by RT‒qPCR and found decreased miR-149-3p levels in CRC tissues compared with normal mucosa tissues (Fig. 4F). Pearson’s correlation analysis revealed that miR-149-3p expression negatively correlated with DDX17 protein level (*R*^2^ = 0.3220, *P* < 0.001, Fig. 4G). Collectively, these results indicate that DDX17 negatively regulated miR-149-3p expression and that miR-149-3p inhibited the migration and invasion of CRC cells.

### CYBRD1 is a direct target of miR-149-3p in CRC cells

**Fig. 5:**
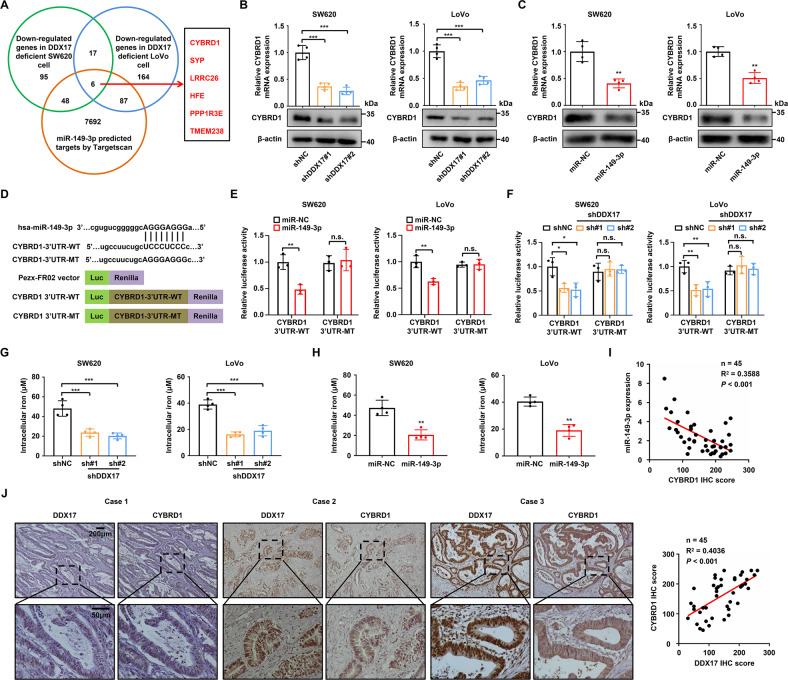
CYBRD1 is a direct target of miR-149-3p in CRC cells. **A** Venn diagram shows the number of downregulated genes identified in response to DDX17 deficiency in SW620 (green) and LoVo (blue) cells with a criterion of 1.5-fold change and adjusted *p* value < 0.05 and predicted miR-149-3p target genes by Targetscan (orange). We identified six common genes among the three groups (red). **B** RT‒qPCR and western blot analysis examined the effects of DDX17 on CYBRD1 expression in SW620 and LoVo cells. **C** RT‒qPCR and western blot analysis examined the impacts of miR-149-3p on CYBRD1 expression in SW620 and LoVo cells. **D** The predicted and mutant miR-149-3p binding sequences in the 3′-UTR of CYBRD1 mRNAs were inserted into the PEZx-FR02 luciferase reporter vector. WT and MT represent the wild type and mutant, respectively. **E** Relative luciferase activity was determined in indicated SW620 and LoVo cells transfected with the wild-type or mutant 3′-UTR of CYBRD1 mRNA. **F** DDX17-deficient SW620 and LoVo cells were transfected with the wild-type or mutant 3′-UTR of CYBRD1 mRNA, and then the relative luciferase activity was determined. **G** The intracellular iron concentration was examined in DDX17-deficient SW620 and LoVo cells. **H** SW620 and LoVo cells were infected with miR-149-3p lentivirus, and then subjected to an iron assay. **I** Correlation analysis between CYBRD1 protein expressions and miR-149-3p levels in CRC tissues. **J** Correlation analysis between the protein expressions of DDX17 and CYBRD1 in CRC tissues. n.s. no significance, **P < 0.01, ***P < 0.001.

To uncover the underlying mechanisms by which the DDX17/miR-149-3p axis controls CRC metastasis, we carried out RNA sequencing and analyzed the downregulated genes triggered by DDX17 with a criterion of 1.5-fold change and adjusted *p* value < 0.05 (Supplementary Tables S2, S3) as well as the predicted targets of miR-149-3p by the TargetScan database. Using this strategy, we identified 6 potential miR-149-3p target genes that were regulated by DDX17 (Fig. 5A). CYBRD1 attracted our special attention, because one previous study has reported that CYBRD1 was associated with CRC carcinogenesis and development [27]. We confirmed that DDX17 knockdown significantly reduced both the mRNA and protein levels of CYBRD1 in SW620 and LoVo cells (Fig. 5B). Similarly, decreased expression of CYBRD1 was observed in miR-149-3p-overexpressing CRC cells (Fig. 5C). Conversely, DDX5 failed to modulate CYBRD1 expression in CRC cells (Supplementary Fig. S8). To explore whether CYBRD1 is a direct target of miR-149-3p, we constructed wild-type or mutant CYBRD1 3′-UTR plasmids based on the PEZx-FR02 luciferase reporter vector (Fig. 5D). Luciferase reporter assays showed that miR-149-3p decreased the luciferase activity by 52% and 37% for the wild-type CYBRD1 3′-UTR in SW620 and LoVo cells, respectively (Fig. 5E). In contrast, there was no obvious inhibitory effect of miR-149-3p on the luciferase activity of the mutant CYBRD1 3′-UTR in CRC cells (Fig. 5E). In addition, the luciferase activity of wild-type CYBRD1 3′-UTR, but not mutant CYBRD1 3′-UTR, was decreased in DDX17-deficient CRC cells compared with control CRC cells (Fig. 5F). Because CYBRD1 is an iron-regulated protein that displays ferric reductase activity and participates in iron absorption in both normal and malignant cells, we subsequently investigated the impact of the DDX17/miR-149-3p axis on intracellular iron abundance in CRC cells. As shown in Fig. 5G, suppression of DDX17 restricted the cellular accumulation of iron ions. MiR-149-3p-overexpressing CRC cells exhibited lower intracellular iron levels than miR-NC CRC cells (Fig. 5H). Pearson’s correlation analysis showed a reverse correlation between CYBRD1 and miR-149-3p in CRC tissues (*R*^2^ = 0.3588, *P* < 0.001, Fig. 5I). Moreover, we found that the protein level of DDX17 was positively associated with CYBRD1 protein expression in CRC samples (*R*^2^ = 0.4036, *P* < 0.001, Fig. 5J). These data indicate that miR-149-3p restrains CYBRD1 expression by binding to the 3′-UTR of CYBRD1 in CRC cells.

### DDX17 accelerates the metastasis and EMT progression of CRC cells through the miR-149-3p/CYBRD1 pathway

**Fig. 6:**
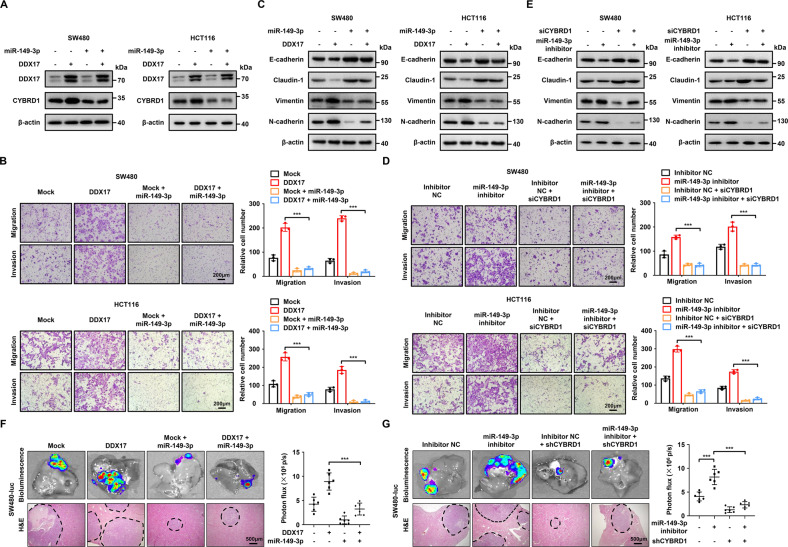
The miR-149-3p/CYBRD1 pathway is required for DDX17-induced CRC cell migration and invasion. **A** Western blot analysis of CYBRD1 expression in SW480 and HCT116 cells infected with DDX17 and/or miR-149-3p lentivirus. **B** Transwell assays in SW480 and HCT116 cells infected with DDX17 and/or miR-149-3p lentivirus. **C** Western blot analysis of EMT marker expression in SW480 and HCT116 cells infected with DDX17 and/or miR-149-3p lentivirus. **D** SW480 and HCT116 cells infected with miR-149-3p inhibitor lentivirus were transfected with or without specific siRNA targeting CYBRD1, and then subjected to transwell assays. **E** SW480 and HCT116 cells infected with miR-149-3p inhibitor lentivirus were transfected with or without specific siRNA targeting CYBRD1, and then subjected to western blot analysis of EMT marker expression. **F** SW480 cells infecting DDX17 lentivirus together with or without miR-149-3p lentivirus were injected into the spleens of nude mice. Representative bioluminescent and H&E staining images of the isolated liver tissues were obtained, and the light emissions were quantified. **G** SW480 cells infecting miR-149-3p inhibitor lentivirus together with or without CYBRD1 shRNA lentivirus were injected into the spleens of nude mice. Representative bioluminescent and H&E staining images of the isolated liver tissues were obtained, and the light emissions were quantified. ***P < 0.001.

To further investigate whether miR-149-3p was a pivotal mediator of the DDX17-induced positive effect on CYBRD1 expression and CRC metastasis, DDX17-overexpressing SW480 and HCT116 cells were infected with miR-149-3p lentivirus. Our results showed that miR-149-3p significantly abrogated the increased level of CYBRD1 caused by DDX17 (Fig. 6A). MiR-149-3p effectively reversed DDX17-triggered elevated migratory and invasive capacities of CRC cells (Fig. 6B). Moreover, miR-149-3p diminished DDX17-induced EMT progression by preventing the reduced expressions of Claudin-1 and E-cadherin and the upregulated levels of Vimentin and N-cadherin (Fig. 6C). Subsequently, we evaluated the functional roles of CYBRD1 in miR-149-3p-mediated CRC metastasis. Transwell assays showed that the miR-149-3p inhibitor enhanced the migration and invasion of CRC cells, which were reversed by CYBRD1 knockdown (Fig. 6D). In addition, CYBRD1 silencing strikingly compromised miR-149-3p inhibitor-induced decreases in Claudin-1 and E-cadherin and increases in Vimentin and N-cadherin levels in SW480 and HCT116 cells (Fig. 6E). The in vivo liver metastatic model showed that DDX17-mediated augmented metastatic abilities of SW480 cells were substantially counteracted by miR-149-3p overexpression (Fig. 6F). We also found that CYBRD1 deficiency decreased miR-149-3p inhibitor-enhanced CRC metastasis in vivo (Fig. 6G). Taken together, these results indicate that miR-149-3p/CYBRD1 signaling is essential for DDX17-mediated CRC metastasis and EMT induction.

## Discussion

Malignant development of CRC is a defined model in which most metastatic features can be acquired during progression from primary adenoma to locally invasive carcinoma over a period of up to thirty years. Once this stage is reached, CRC cells can rapidly disseminate and colonize distant organs without latency, and the liver is the most common site of metastatic CRC [28, 29]. Hence, identifying specific biomarkers will help us to more precisely evaluate the risk of CRC metastasis. In the present study, we provided the first evidence that higher level of DDX17 was found in CRC liver metastasis tissues than in patient-matched primary CRC tissues. Moreover, DDX17 expression was positively associated with lymph node invasion and advanced AJCC stage. Upregulation of DDX17 facilitated cell migration and invasion, whereas DDX17 suppression attenuated the motility of CRC cells. Importantly, DDX17 was an independent factor for predicting poor prognosis in patients with CRC. Consistent with our results, a recent research discovered that DDX17 was increased in glioma tissues and associated with worse clinical outcome of glioma patients. This study also found a correlation between DDX17 level and sensitivity to radiotherapy or chemotherapy [30]. Our previous study demonstrated that DDX17 contributed to gefitinib resistance by promoting nuclear translocation and activation of β-catenin in non-small cell lung cancer [23]. Moreover, DDX17 was one of the hub genes associated with acquired resistance to cisplatin and fluorouracil combination-based chemotherapy in gastric cancer patients [31]. Based on the above findings, we propose that DDX17 is a valuable predictor of metastasis risk and prognosis for CRC patients, and the assessment of DDX17 expression may be beneficial for the development of a personalized therapeutic strategy.

DDX17 is an important component of the Drosha Microprocessor and can enhance pri-miRNA processing in numerous cellular contexts. In the present study, we identified 126 miRNAs downregulated by DDX17 suppression in SW620 cells, including miR-190b, miR-542-5p and miR-301a-3p. MiR-190b exhibited an oncogenic function in CRC and bladder cancer [32, 33]. Increased expression of miR-542-5p was observed in the plasma of CRC patients compared with non-CRC subjects, and was associated with abnormal purine metabolism [34]. Overexpression of miR-301a-3p accelerated the growth and metastasis of CRC cells by targeting DLC-1 and RUNX3 [35]. These studies combined with our present data further support a critical role of DDX17 in CRC malignant progression. Although miR-149-3p was the most upregulated miRNA by DDX17 deficiency, DDX17 failed to alter the expressions of pri-miR-149, pre-miR-149 and miR-149-5p. Moreover, we did not find a direct interaction between pri-miR149 and DDX17. Mori and colleagues demonstrated that DDX17 recognized and bound to a functional motif (VCAUCH) in the 3′ flanking region of a subset of pri-miRNAs, resulting in the efficient cleavage from pri-miRNAs into pre-miRNAs [24]. Consistently, we found a lack of this functional motif in the pri-miR149 sequence, suggesting that miR-149-3p is an indirect target of DDX17. How DDX17 modulates miR-149-3p expression needs to be clarified in future studies.

DDX5 is a close homolog of DDX17 and can interact with DDX17 to form heterodimers. Thus, DDX5 emerges to have functional redundancy with DDX17 in various physiological and pathological processes. For example, DDX5 and DDX17 dynamically orchestrate the alternative splicing of exons and transcriptional programs of myogenesis-related genes during cell differentiation [36]. Co-suppression of DDX5 and DDX17 remarkably blocks DNA replication and induces cell death by preventing rRNA biogenesis [37]. Although DDX5 has been shown to be highly expressed in CRC tissues and to augment the anchorage-independent growth and metastasis of CRC cells [38, 39], we observed no significant difference in miR-149-3p and CYBRD1 levels in DDX5-deficient CRC cells compared with control cells. Therefore, we infer that DDX5 and DDX17 have distinct nonoverlapping downstream effectors to accelerate CRC development. Consistent with our findings, a recent study reported that DDX17, but not DDX5, restricts the replication of Rift Valley fever virus in human cells [40]. Polyubiquitination of DDX17 at Lys190 mediated by the E3 ligase HectH9 under hypoxia interacts with YAP and p300 to enhance the transcription of stemness-related genes and tumor-initiating capabilities in head and neck squamous cell carcinoma, while DDX5 does not contain the Lys residue corresponding to Lys190 on DDX17 and consequently fails to bind to YAP [41]. The tyrosine phosphorylation of DDX5 at Y593 caused by c-Abl contributes to the coactivation of androgen receptor and tumorigenesis in prostate cancer. However, DDX17 is unable to activate AR-dependent transcription either alone or in combination with DDX5 [42]. These data indicate that the posttranslational modification of DDX5 and DDX17 should be considered to accurately assess their functions in various cellular processes.

Accumulating evidence indicates that miR-149-3p acts as an oncogene or tumor suppressor in different types of cancers. For instance, miR-149-3p augmented tumor growth and the spread of cells by downregulating the expression of BAD2IP [43]. MiR-149-3p was increased in NSCLC tissues and accelerated malignant development of non-small cell lung cancer cells through blockage of KLLN-mediated p53 activation [44]. In contrast, miR-149-3p triggered apoptosis and suppressed the tumorigenicity of spinal chordoma cells by targeting the Smad3 gene [45]. MiR-149-3p prevented regulatory T-cell differentiation and immune escape via posttranscriptional inhibition of FOXP3 mRNA in esophageal cancer [46]. Here, our data revealed that miR-149-3p was decreased in CRC tissues and negatively regulated the metastasis of CRC cells, suggesting a suppressive role of miR-149-3p in CRC development. We demonstrated that CYBRD1 was a direct target of miR-149-3p and was essential for miR-149-3p-mediated motility of CRC cells. We also found a negative correlation between CYBRD1 and miR-149-3p in CRC tissues. In line with our observation, one study reported that iron levels and the expression of iron import proteins (including CYBRD1, divalent metal transporter 1, and transferrin receptor 1) were increased in CRC tissues, and iron loading promoted proliferation and inhibited cell adhesion in CRC cells [27]. Ferroptosis is a recently identified nonapoptotic cell death characterized by an iron-catalyzed excessive peroxidation of polyunsaturated fatty acid-containing phospholipids, leading to the instability and permeabilization of the cell membrane. As cancer cells exhibit higher iron requirements than normal cells (termed “iron addiction”) [47, 48], CRC cells may be more sensitive and vulnerable to ferroptosis. Therefore, treatment with ferroptosis-inducing compounds may be a promising strategy for CRC therapy.

**Fig. 7:**
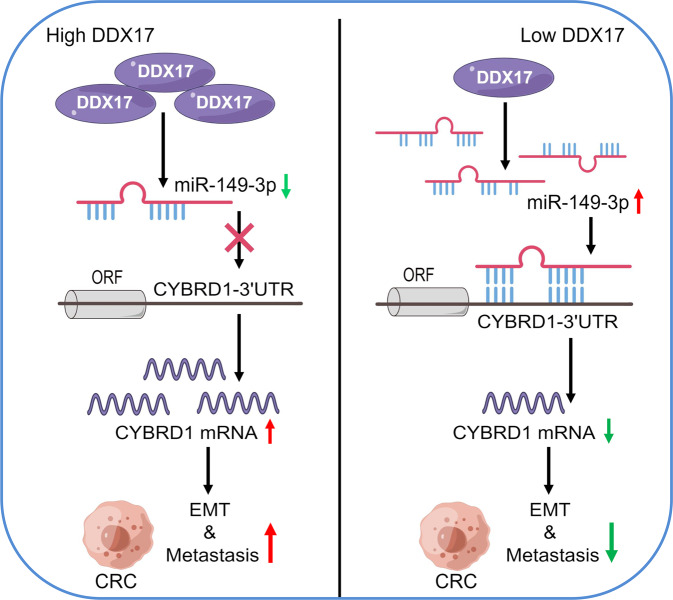
Schematic diagram of the DDX17/miR-149-3p/CYBRD1 signaling pathway in the modulation of EMT and CRC progression. DDX17 is elevated in CRC and decreases the expression of miR-149-3p independent of DDX17’s Microprocessor function. Downregulated miR-149-3p attenuates its binding to the 3′-UTR of CYBRD1 mRNA, leading to increases CYBRD1 expression and intracellular iron level, which promotes EMT progression and metastasis of CRC cells.

In summary, our current study provides the first evidence that upregulated DDX17 expression is associated with aggressive phenotypes and poor prognosis in CRC. We demonstrated that DDX17 augmented CRC cell metastasis and EMT progression via downregulation of miR-149-3p, which resulted in increased CYBRD1 level by decreased binding to the 3′-UTR of CYBRD1 mRNA (Fig. 7). Taken together, our findings highlight the significance of DDX17 in the clinical outcome of CRC patients and delineate an important molecular mechanism by which DDX17 contributes to CRC metastasis through manipulation of the miR-149-3p/CYBRD1 axis. Thus, the DDX17/miR-149-3p/CYBRD1 signaling pathway may represent a promising therapeutic strategy for the treatment of CRC.

## Supplementary information

Reproducibility checklist

Author Contribution Statement

Confirmation of the order of contributing authors

Supplementary Figure legend

Supplementary Table S1

Supplementary Table S2

Supplementary Table S3

Supplemental Figure S1

Supplemental Figure S2

Supplemental Figure S3

Supplemental Figure S4

Supplemental Figure S5

Supplemental Figure S6

Supplemental Figure S7

Supplemental Figure S8

Original western blots

## Data Availability

All data are available within the main article, supplementary materials, or available from the corresponding author upon reasonable request.
