# Supplementary material for: DDX17 induces epithelial-mesenchymal transition and metastasis through the miR-149-3p/CYBRD1 pathway in colorectal cancer
Source: Cell Death Dis. 2023 Jan 2;14(1):1. doi: 10.1038/s41419-022-05508-y (PMC9807641; doi:10.1038/s41419-022-05508-y)
Supplement: Supplementary file 2 — Author Contribution Statement [file 41419_2022_5508_MOESM2_ESM.docx]

**Author Contribution Statement**

**Gang Zhao:** Data curation, Formal analysis, Funding acquisition, Investigation, Writing - review & editing. **Qijing Wang:** Data curation, Formal analysis, Investigation. **Yue Zhang:** Data curation, Formal analysis, Investigation. **Rui Gu:** Formal analysis, Investigation. **Min Liu:** Formal analysis, Methodology, Validation. **Qin Li:** Resources, Software. **Jie Zhang:** Resources, Software. **Hang Yuan:** Data curation, Validation. **Tianyu Feng:** Data curation, Validation. **Deqiong Ou:** Methodology, Visualization. **Siqi Li:** Methodology, Visualization. **Shan Li:** Methodology. **Kai Li:** Data curation, Formal analysis, Funding acquisition, Investigation. **Chunfen Mo:** Conceptualization, Methodology, Software, Funding acquisition, Writing - original draft. **Ping Lin:** Conceptualization, Funding acquisition, Project administration, Supervision, Writing - review & editing.

All authors have read and approved the final version of this article.
