# Supplementary material for: DDX17 induces epithelial-mesenchymal transition and metastasis through the miR-149-3p/CYBRD1 pathway in colorectal cancer
Source: Cell Death Dis. 2023 Jan 2;14(1):1. doi: 10.1038/s41419-022-05508-y (PMC9807641; doi:10.1038/s41419-022-05508-y)
Supplement: Supplementary file 4 — Supplementary Figure legend [file 41419_2022_5508_MOESM4_ESM.docx]

**Supplementary Figure S1. Both mRNA and protein levels of DDX17 are increased in CRC.** (A) DDX17 mRNA expression in colon adenocarcinoma and normal tissues from the TCGA database. (B) DDX17 protein expression in primary colon cancer and normal tissues from the CPTAC database.

**Supplementary Figure S2. Expression of DDX17 in CRC cell lines.** (A) Western blot analysis of DDX17 expression in seven CRC cell lines. (B) Relative DDX17 mRNA levels in a series of CRC cell lines from the CCLE dataset.

**Supplementary Figure S3. DDX17 promotes the migration and invasion of CRC cell *in vitro* and *in vivo*.** (A-B) RT-qPCR (A) and western blot (B) analysis verified the upregulation of DDX17 in SW480 and HCT116 cells. (C) Microscopic observations were recorded at 0, 24, 48 and 72 hours after scratching the surface of a confluent layer of indicated SW480 and HCT116 cells. (D) The effects of DDX17 on cell migration and invasion were examined by transwell assay in SW480 and HCT116 cells. (E) The numbers of adhesive cells on the Matrigel were recorded after 30, 60, 90, and 120 mins in indicated SW480 and HCT116 cells. (F) DDX17 overexpressing and mock SW480 cells were injected into the spleens of nude mice. Representative bioluminescent and H&E staining images of the isolated liver tissues were obtained, and the light emissions were quantified. n.s. no significance, **P* < 0.05, ***P* < 0.01, ****P* < 0.001.

**Supplementary Figure S4. DDX17 suppression attenuates the proliferation of CRC cells.** (A-B) The cell viability (A) and anchorage-dependent cell growth ability (B) of DDX17-knockdown CRC cells were measured by CCK8 assays or colony formation assays. ***P* < 0.01.

**Supplementary Figure S5. RT-qPCR analysis determined the impact of DDX5 on miR-149-3p expression in SW620 and LoVo cells.** n.s. no significance, ****P* < 0.001.

**Supplementary Figure S6. DDX17 regulated miR-149-3p expression independent of its Microprocessor function.** (A) Pri-miR-149 and pri-miR-21 were *in vitro* transcripted and biotinylated. Biotinylated pri-miR-149 and pri-miR-21 were incubated with protein lysates from SW620 cells, and then RNA-protein binding assay was performed. (B) RT-qPCR analysis examined the effects of DDX17 on the expressions of pri-miR-149, pre-miR-149 and miR-149-5p in SW620 and LoVo cells. n.s. no significance, ****P* < 0.001.

**Supplementary Figure S7. RT-qPCR analysis verified the upregulation of miR-149-3p in SW620 and LoVo cells infected with miR-149-3p lentivirus.** ****P* < 0.001.

**Supplementary Figure S8. RT-qPCR and western blot analysis examined the effect of DDX5 on CYBRD1 expression in SW620 and LoVo cells.** n.s. no significance.
