# Supplementary figures and images for: DDX17 induces epithelial-mesenchymal transition and metastasis through the miR-149-3p/CYBRD1 pathway in colorectal cancer
Source: Cell Death Dis. 2023 Jan 2;14(1):1. doi: 10.1038/s41419-022-05508-y (PMC9807641; doi:10.1038/s41419-022-05508-y)

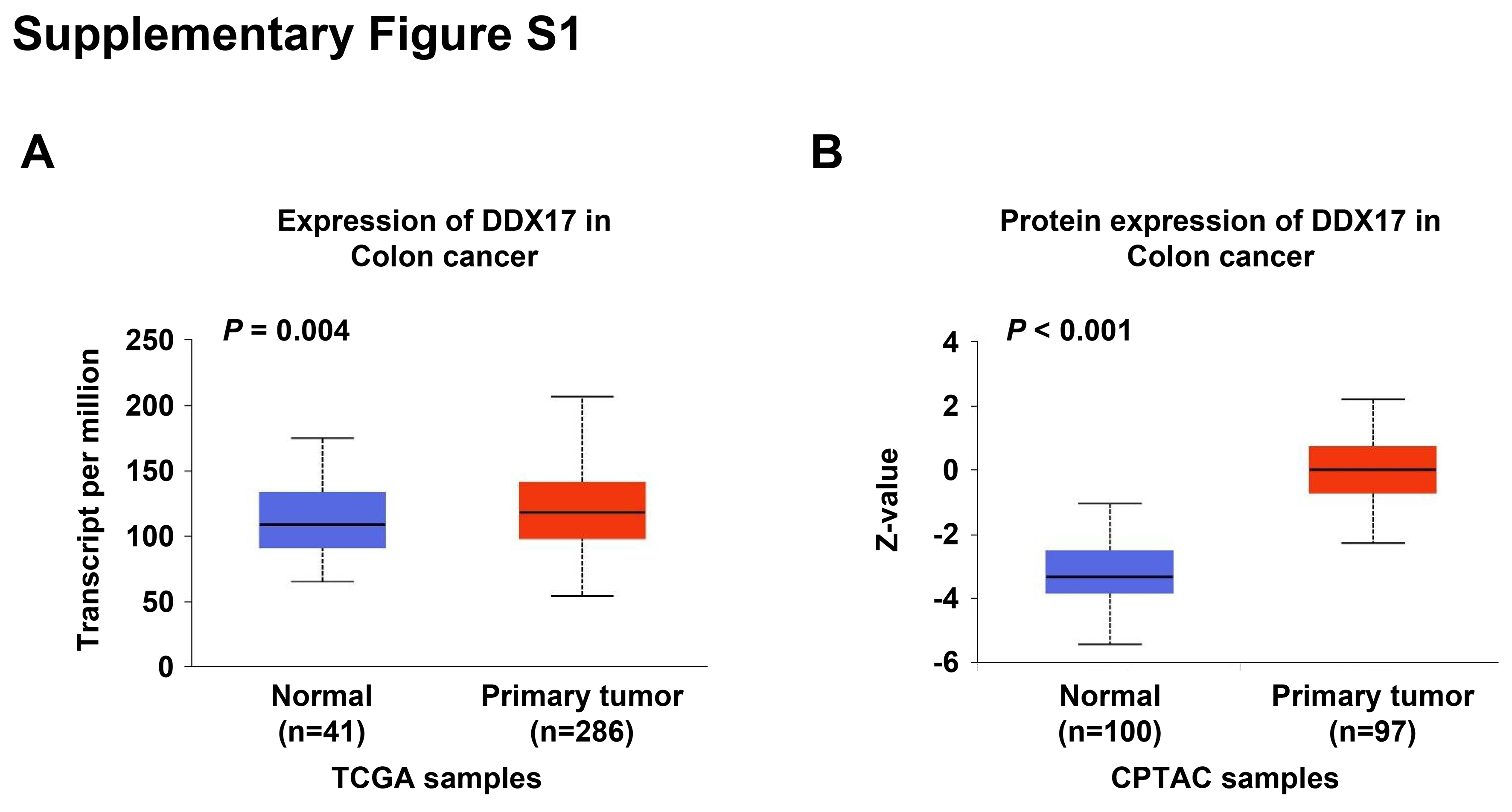

Supplement: Supplementary file 8 — Supplemental Figure S1 [file 41419_2022_5508_MOESM8_ESM.tif]

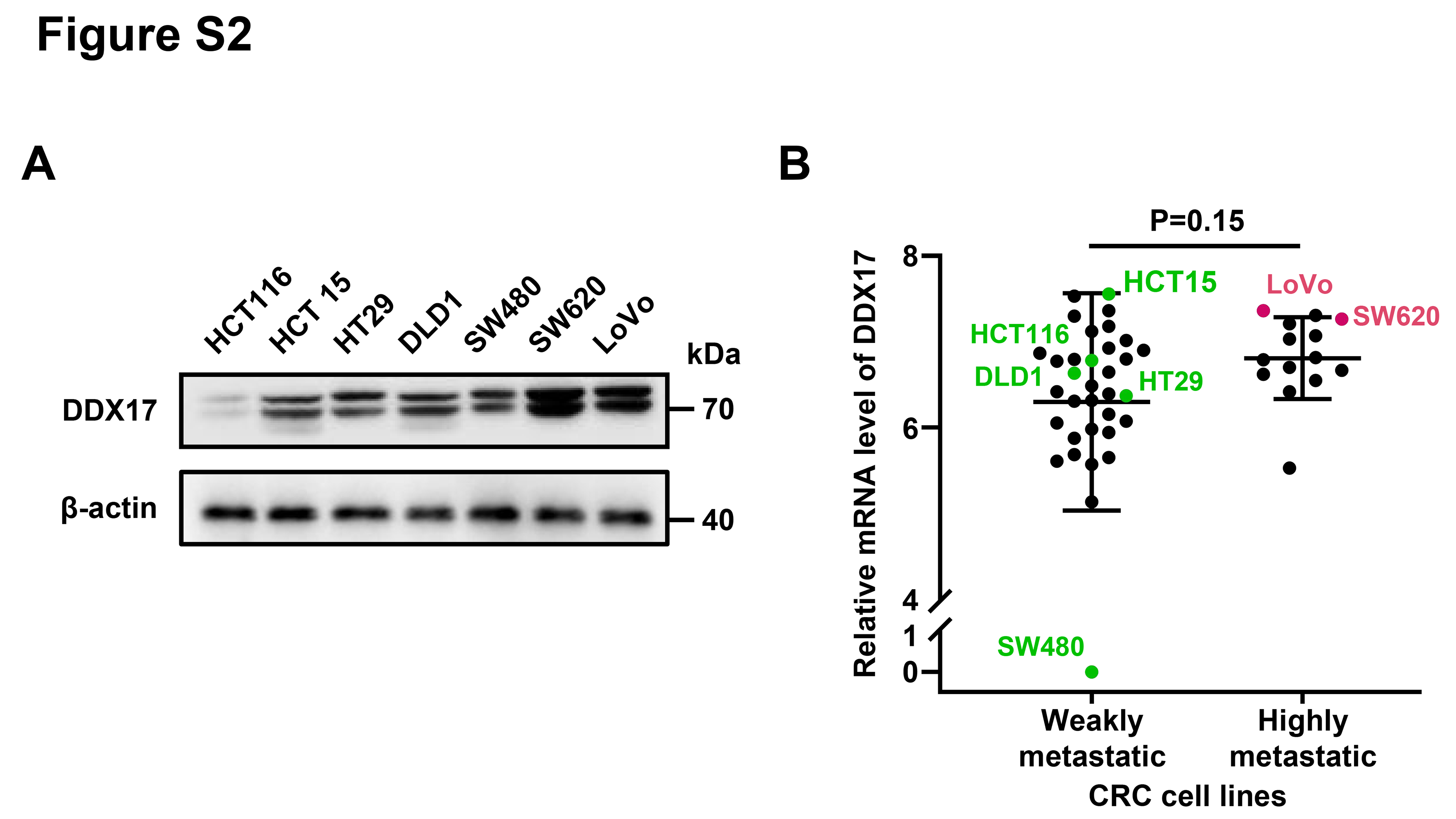

Supplement: Supplementary file 9 — Supplemental Figure S2 [file 41419_2022_5508_MOESM9_ESM.tif]

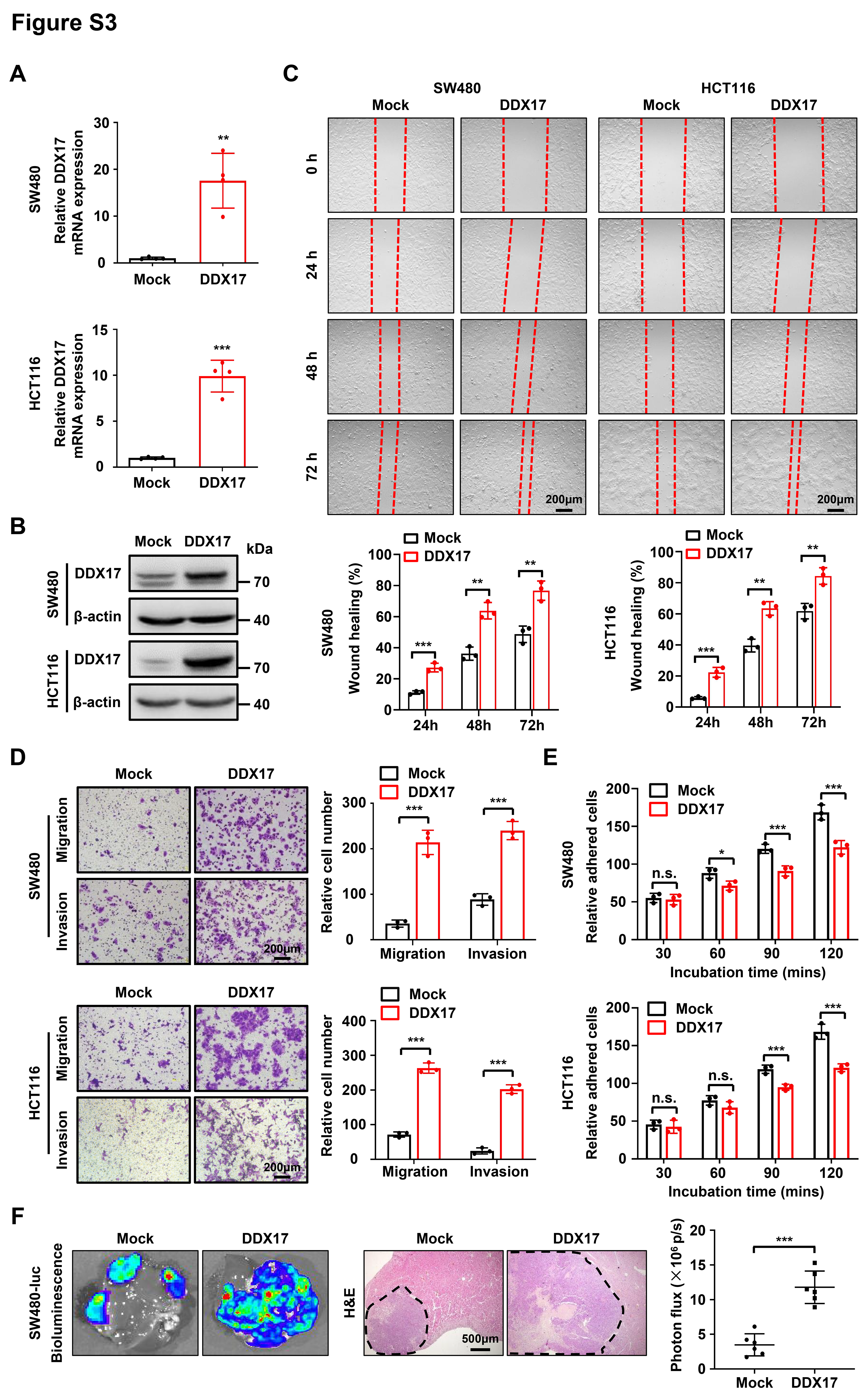

Supplement: Supplementary file 10 — Supplemental Figure S3 [file 41419_2022_5508_MOESM10_ESM.tif]

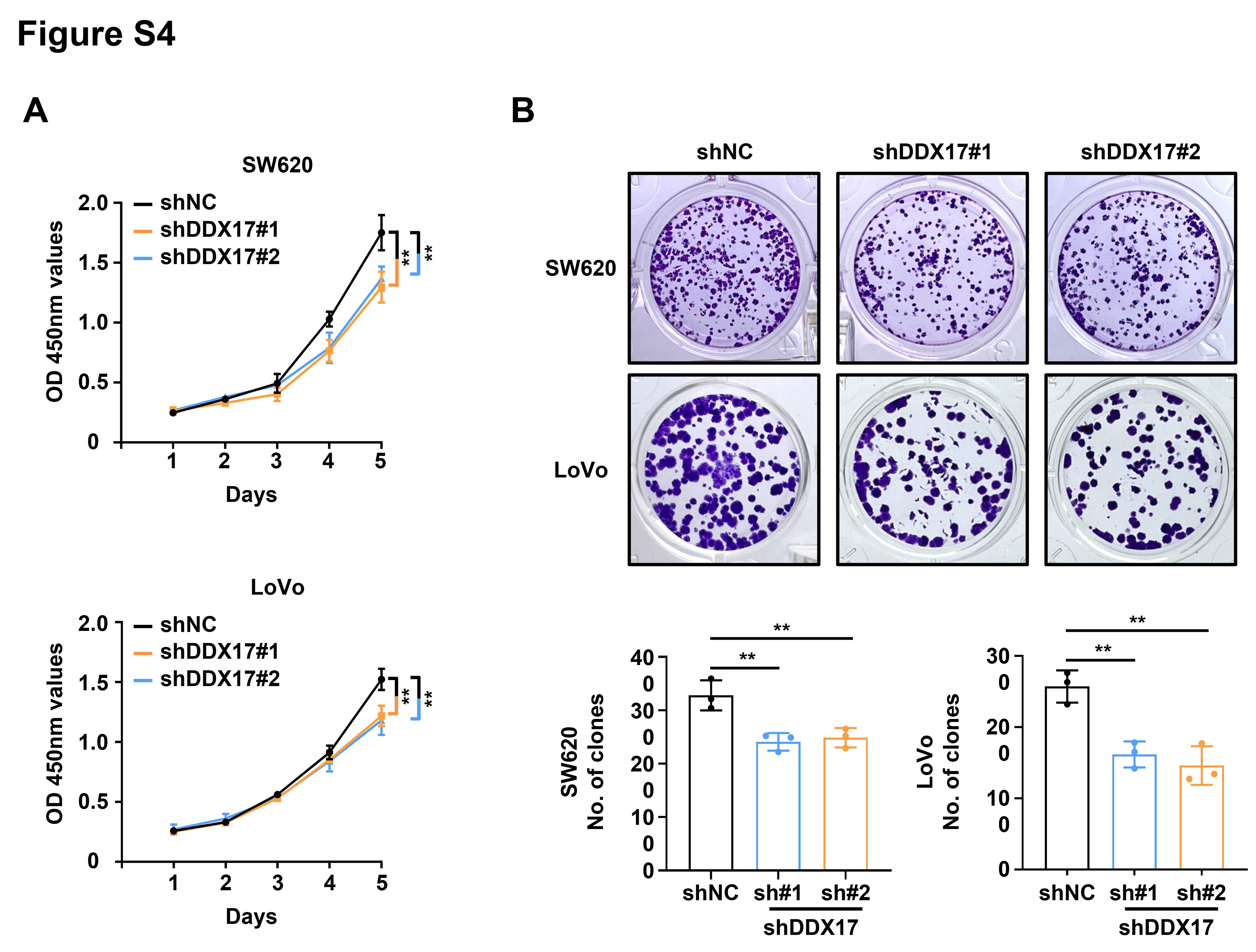

Supplement: Supplementary file 11 — Supplemental Figure S4 [file 41419_2022_5508_MOESM11_ESM.tif]

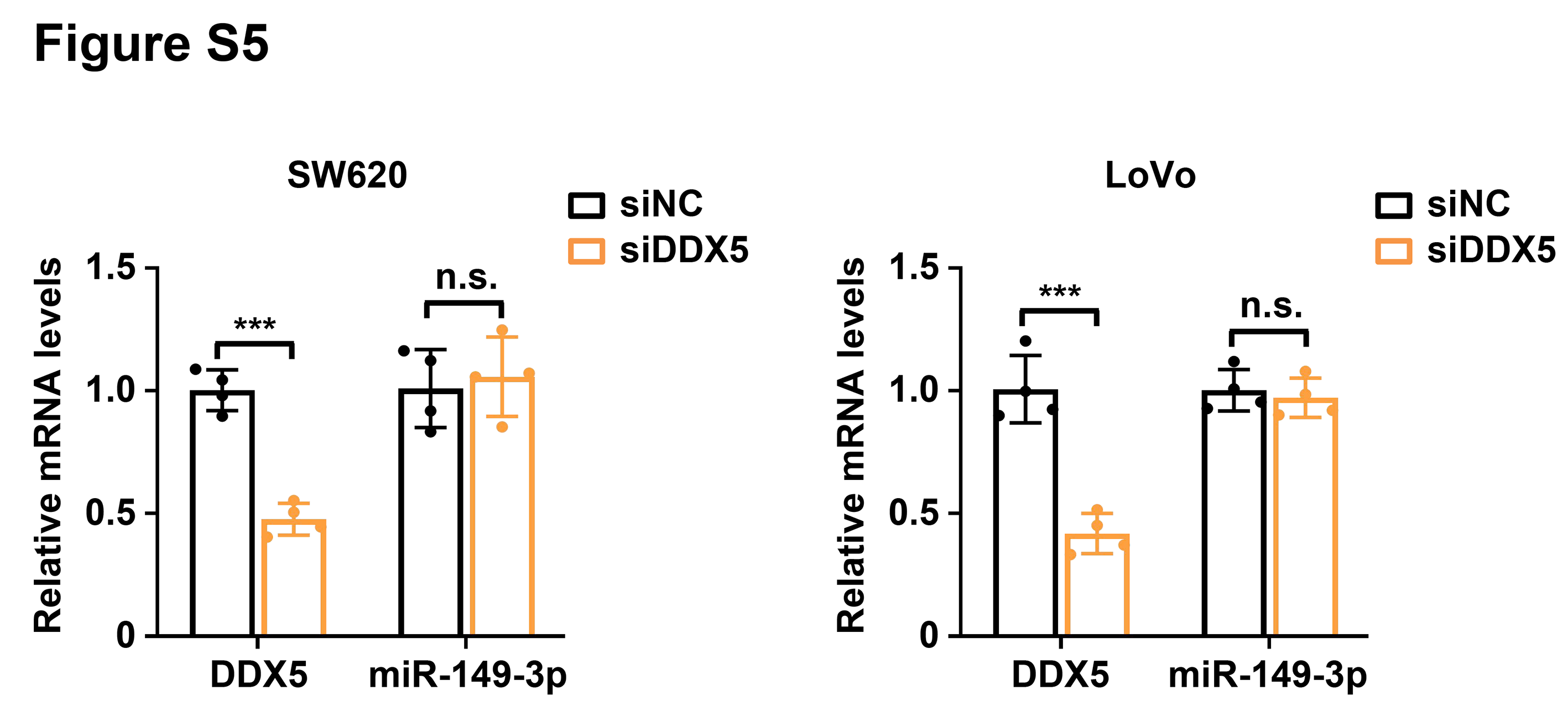

Supplement: Supplementary file 12 — Supplemental Figure S5 [file 41419_2022_5508_MOESM12_ESM.tif]

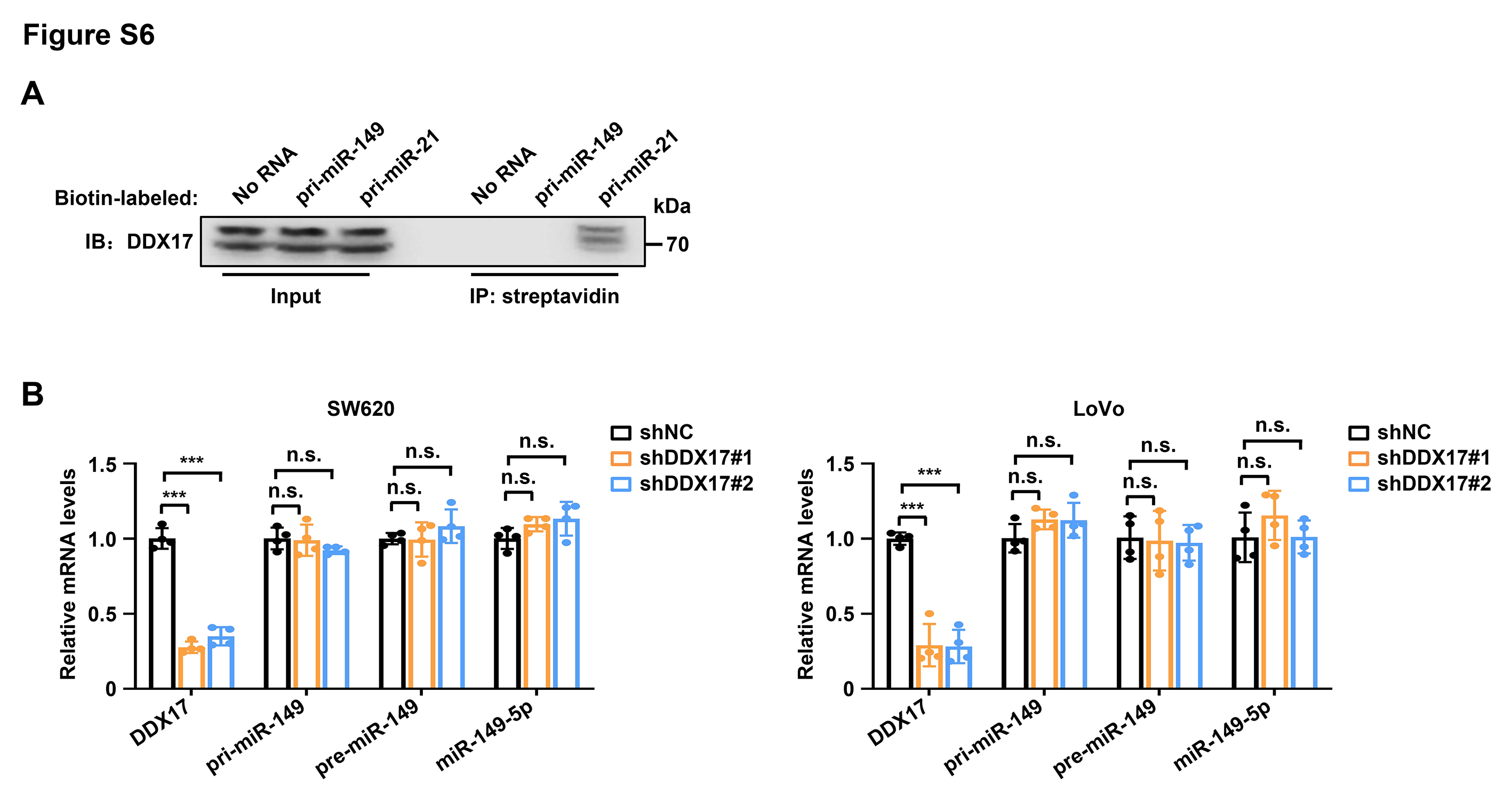

Supplement: Supplementary file 13 — Supplemental Figure S6 [file 41419_2022_5508_MOESM13_ESM.tif]

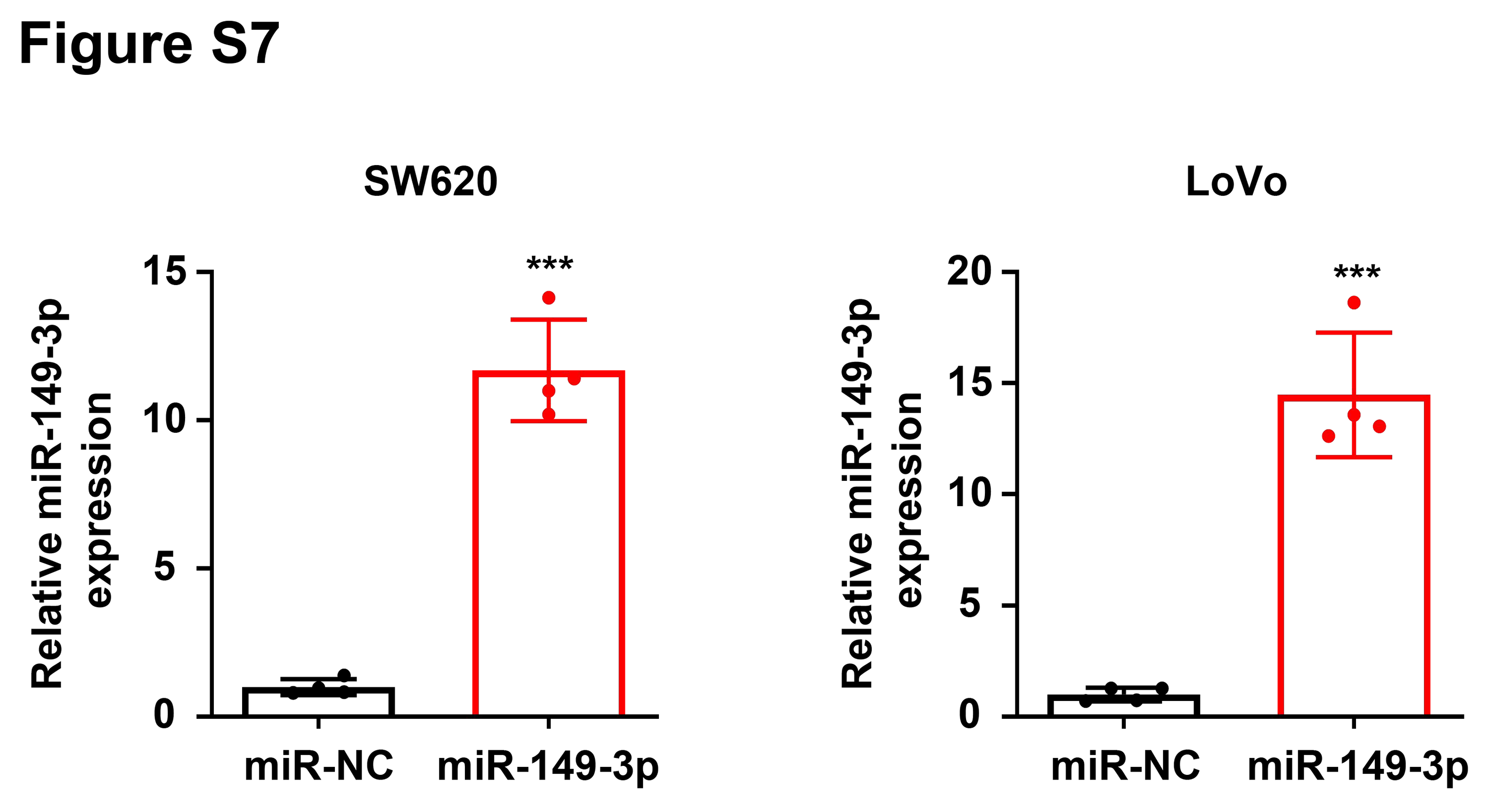

Supplement: Supplementary file 14 — Supplemental Figure S7 [file 41419_2022_5508_MOESM14_ESM.tif]

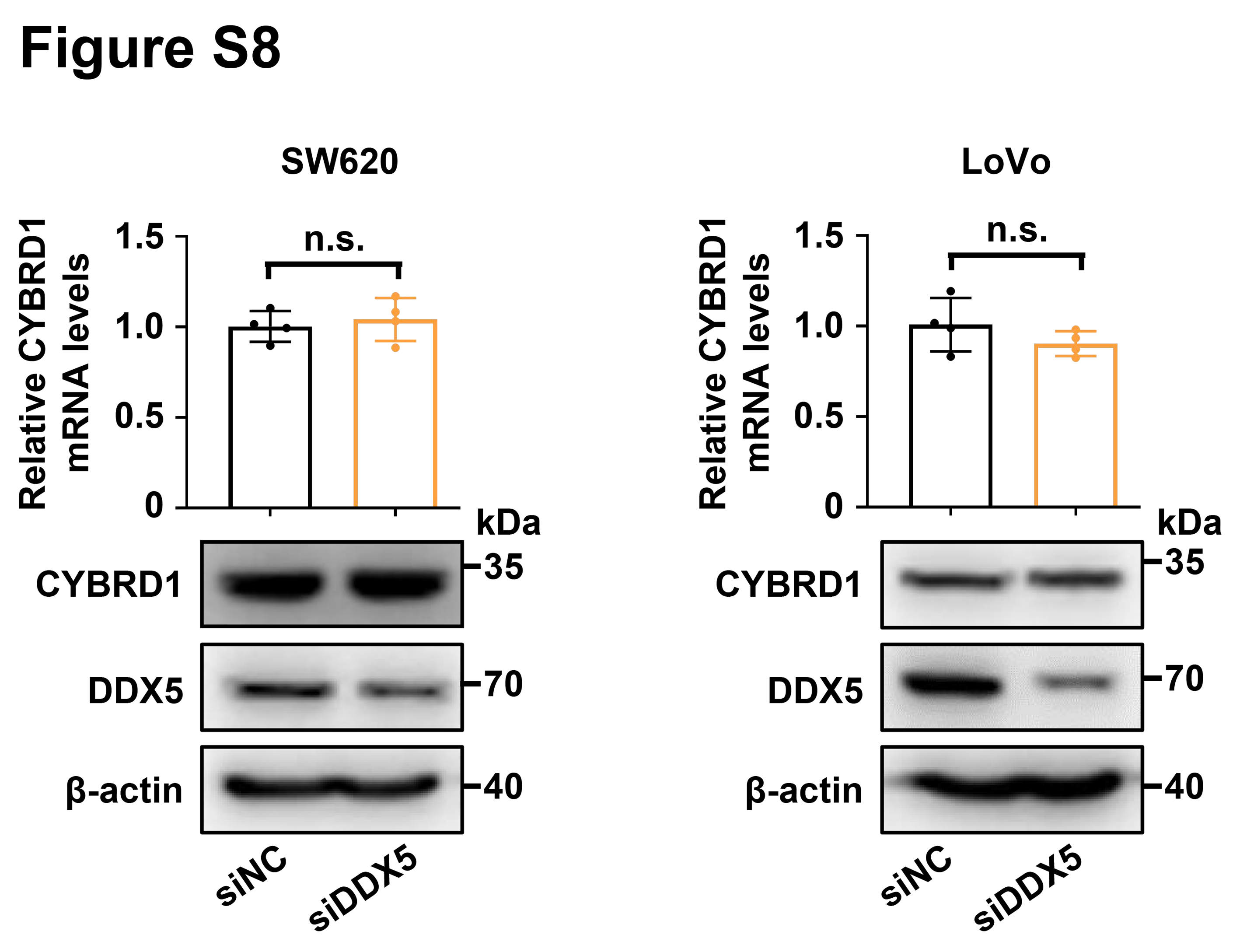

Supplement: Supplementary file 15 — Supplemental Figure S8 [file 41419_2022_5508_MOESM15_ESM.tif]

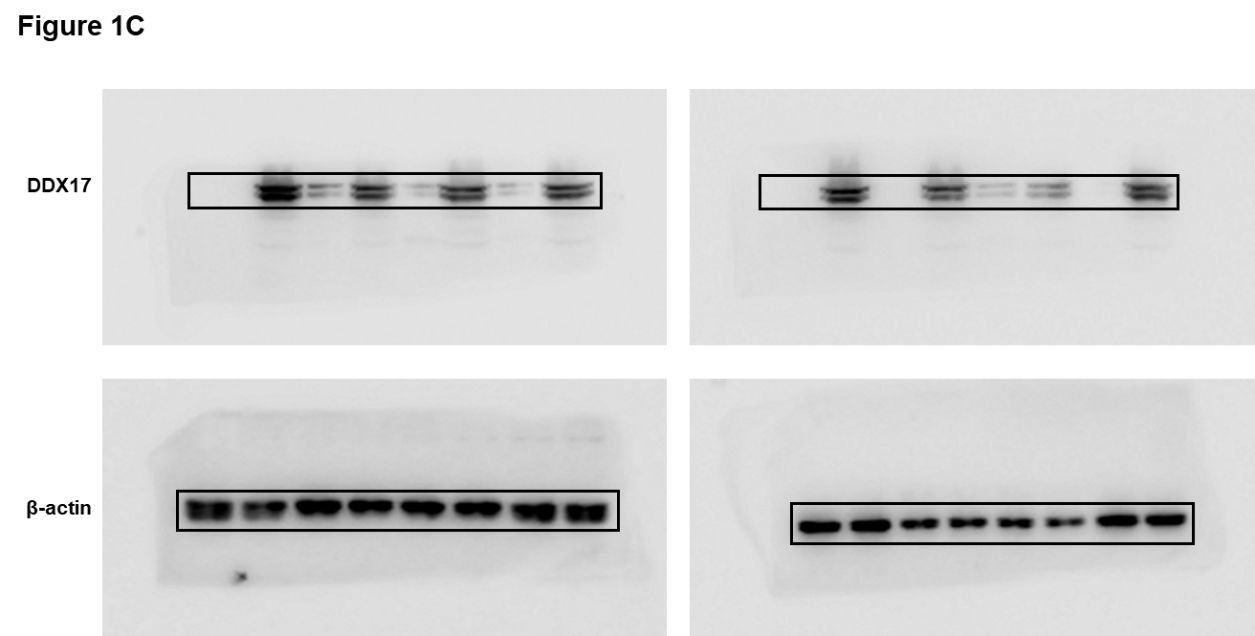


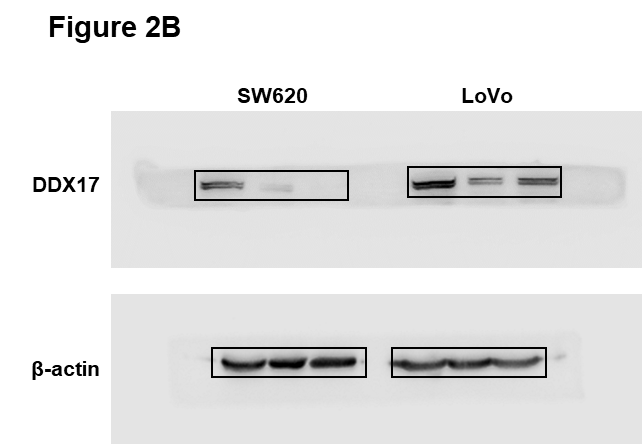


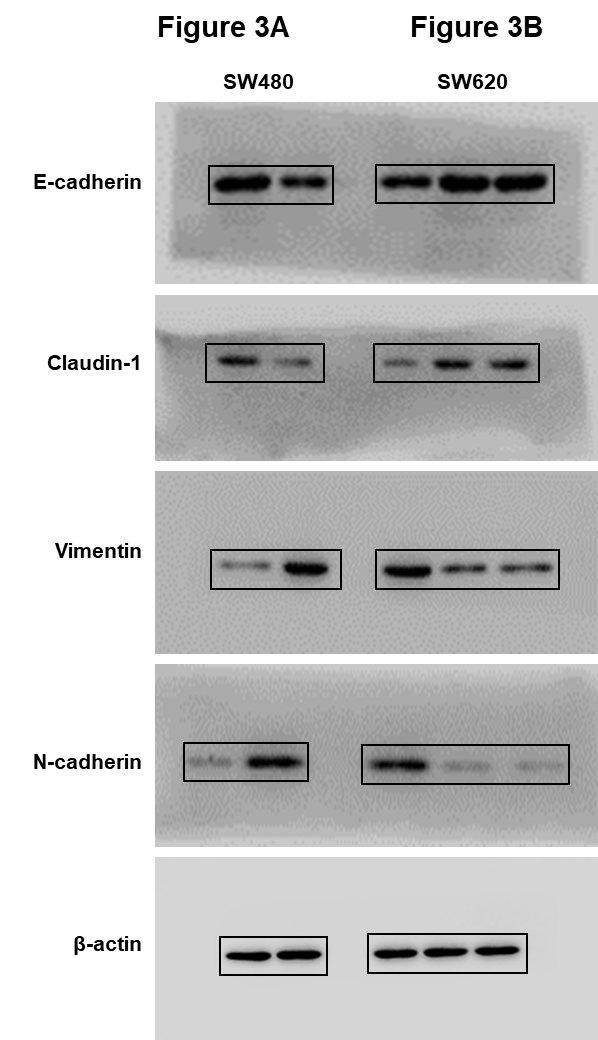


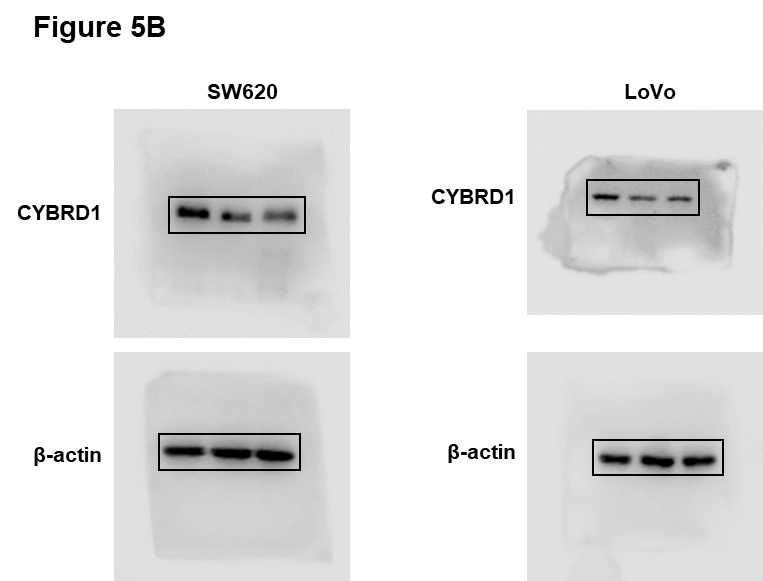


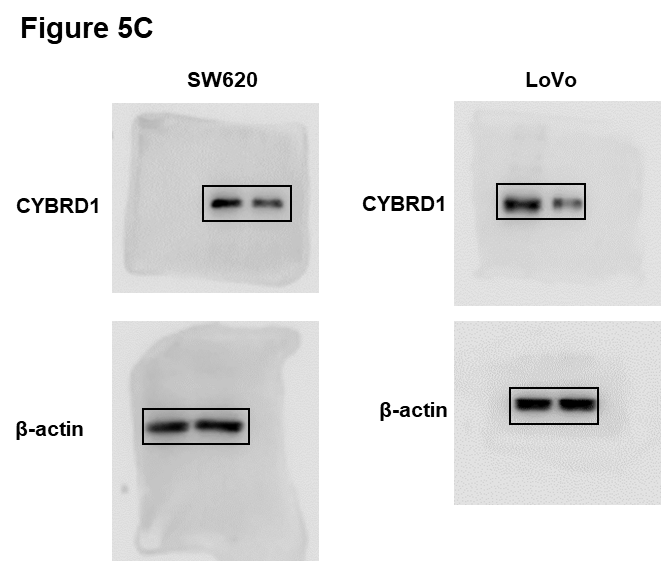


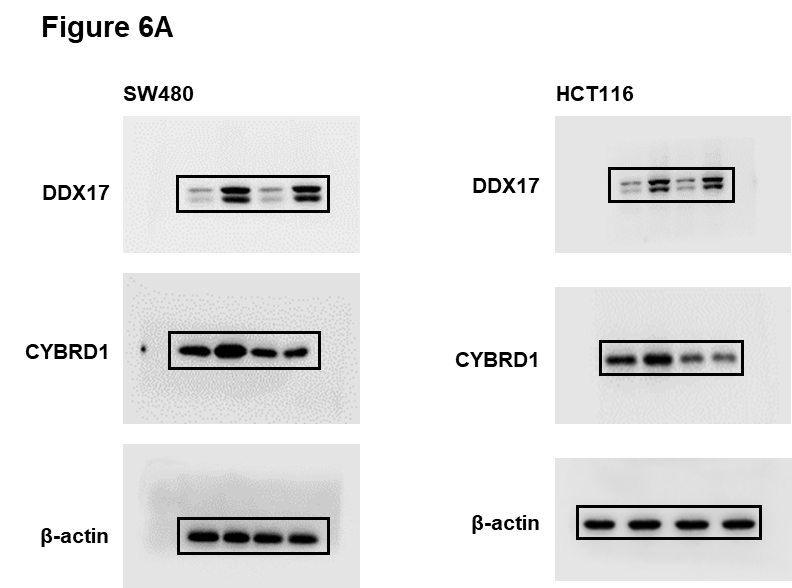


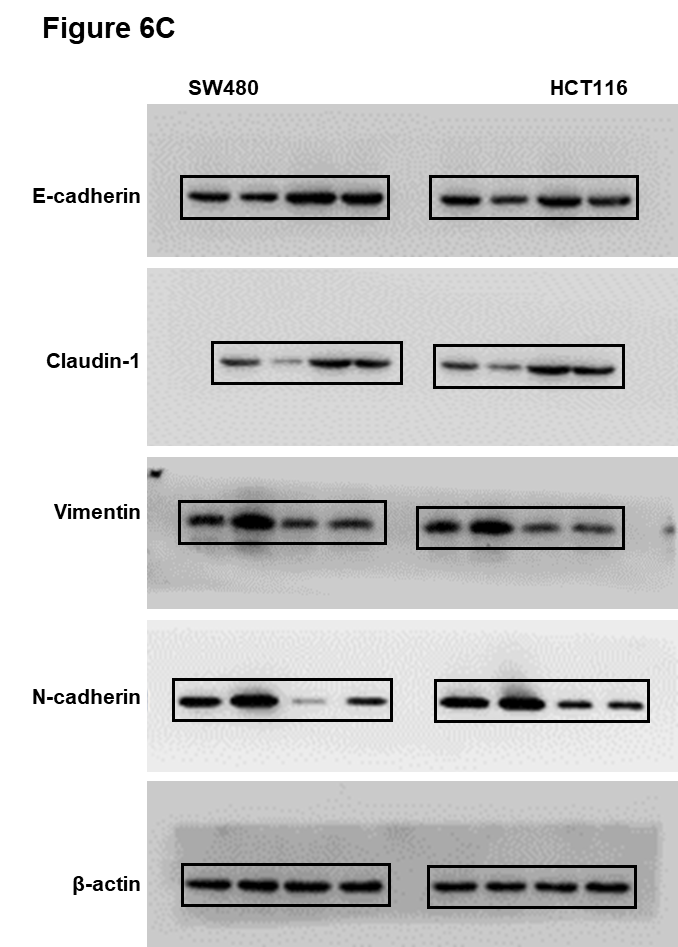


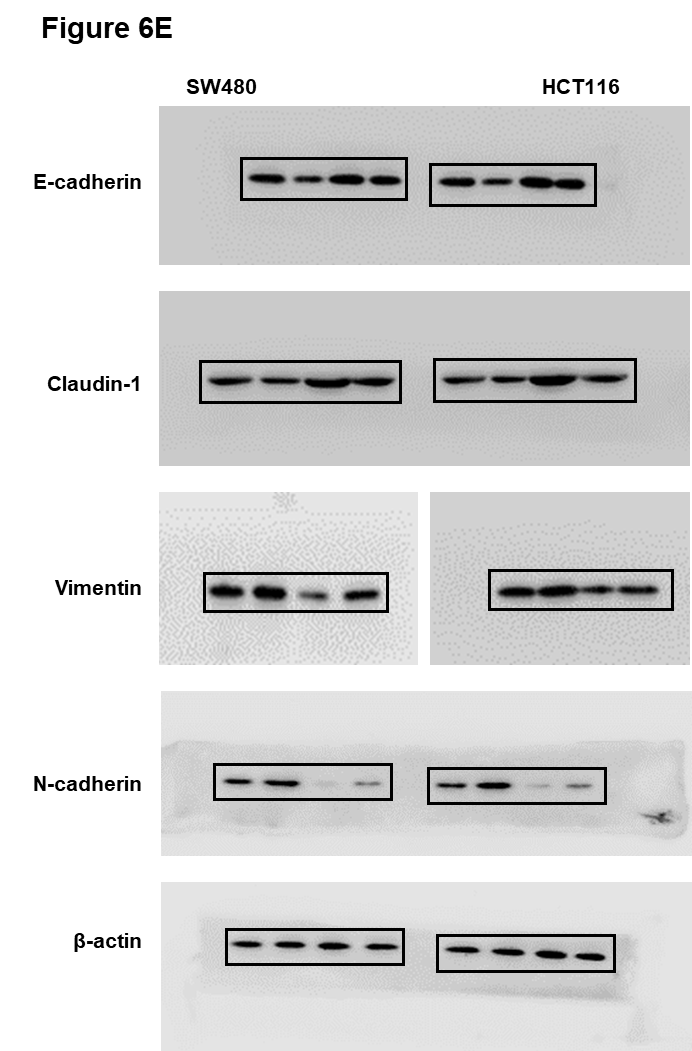


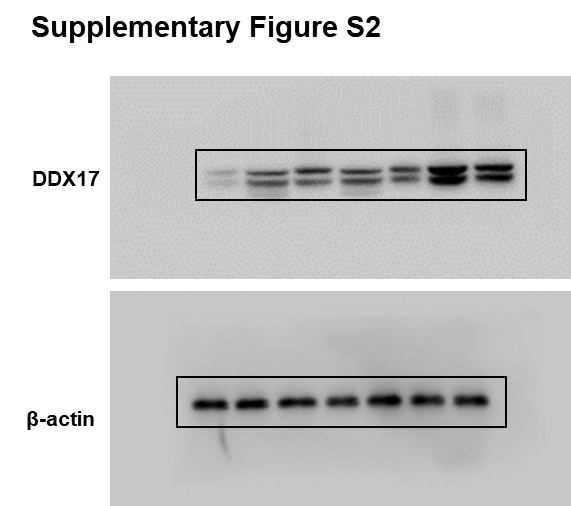


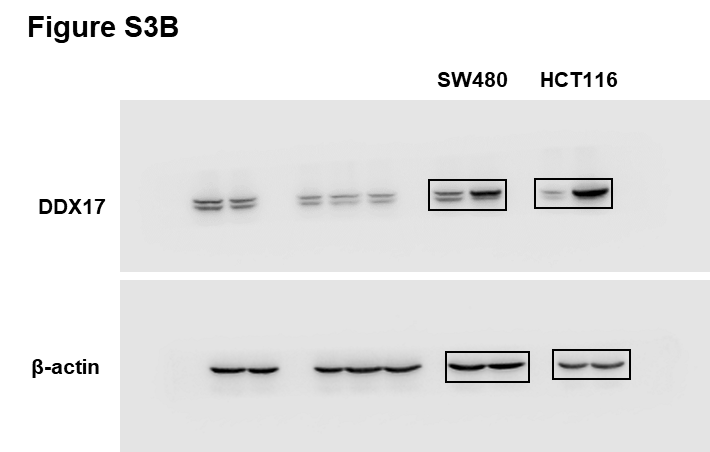


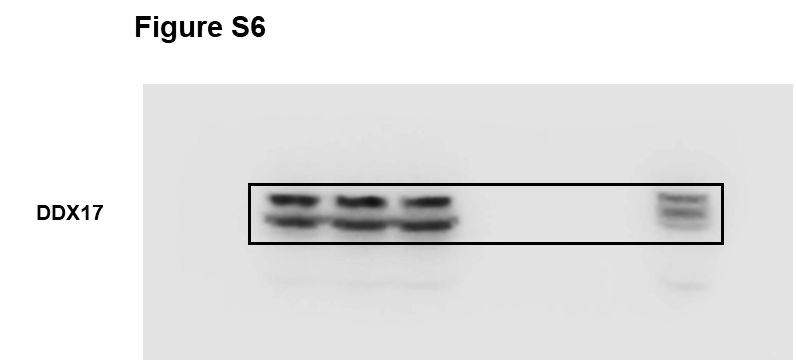


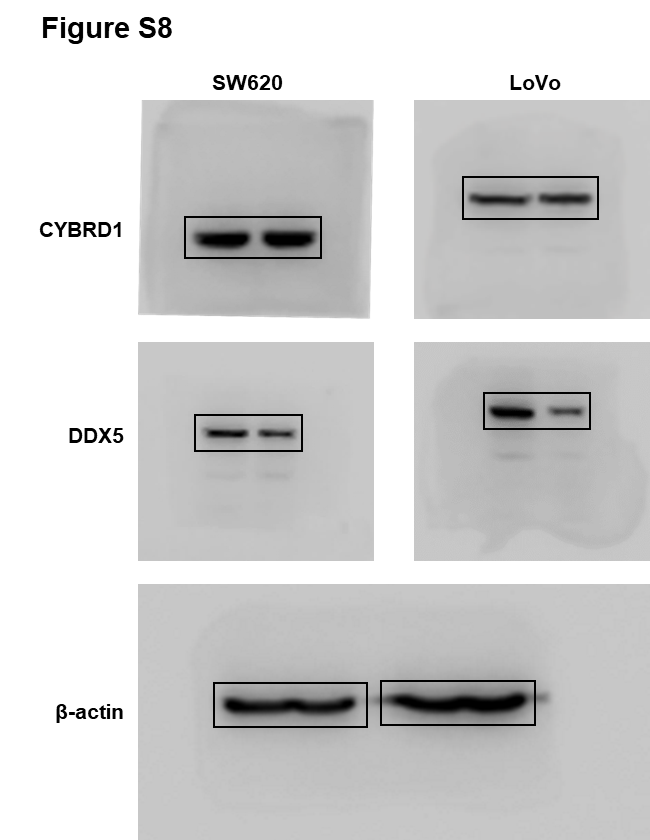

Supplement: Supplementary file 16 — Original western blots [file 41419_2022_5508_MOESM16_ESM.docx]
