# Supplementary material for: DDX17 induces epithelial-mesenchymal transition and metastasis through the miR-149-3p/CYBRD1 pathway in colorectal cancer
Source: Cell Death Dis. 2023 Jan 2;14(1):1. doi: 10.1038/s41419-022-05508-y (PMC9807641; doi:10.1038/s41419-022-05508-y)
Supplement: Supplementary file 3 — Confirmation of the order of contributing authors [file 41419_2022_5508_MOESM3_ESM.docx]

Editorial Office

Cell Death & Disease

December 7, 2022

Dear Editor:

I have emailed our co-authors with the change, and they all agreed and confirmed the final version of manuscript (number: CDDIS-22-3194RR) and the order of contributing authors in this manuscript. The screenshot of their replies is attached below.

Please contact me if any questions arise. Thank you very much!

Sincerely,

Ping Lin, Professor

Lab of Experimental Oncology, State Key Laboratory of Biotherapy and Cancer Center,

and Frontiers Science Center for Disease-related Molecular Network,

West China Hospital, Sichuan University,

Chengdu, P.R. China, 610041

Tel: +86-28-85164016

Fax: +86-28-85582944

E-mail: linping@scu.edu.cn

Screenshot of the contributing authors’ replies:
